# Prostaglandins limit nuclear actin to control nucleolar function during oogenesis

**DOI:** 10.3389/fcell.2023.1072456

**Published:** 2023-02-17

**Authors:** Danielle E. Talbot, Bailey J. Vormezeele, Garrett C. Kimble, Dylane M. Wineland, Daniel J. Kelpsch, Michelle S. Giedt, Tina L. Tootle

**Affiliations:** Anatomy and Cell Biology, University of Iowa Carver College of Medicine, Iowa City, IA, United States

**Keywords:** prostaglandins, nucleolus, nuclear actin, oogenesis, *Drosophila* prostaglandins limit nucleolar function

## Abstract

Prostaglandins (PGs), locally acting lipid signals, regulate female reproduction, including oocyte development. However, the cellular mechanisms of PG action remain largely unknown. One cellular target of PG signaling is the nucleolus. Indeed, across organisms, loss of PGs results in misshapen nucleoli, and changes in nucleolar morphology are indicative of altered nucleolar function. A key role of the nucleolus is to transcribe ribosomal RNA (rRNA) to drive ribosomal biogenesis. Here we take advantage of the robust, *in vivo* system of *Drosophila* oogenesis to define the roles and downstream mechanisms whereby PGs regulate the nucleolus. We find that the altered nucleolar morphology due to PG loss is not due to reduced rRNA transcription. Instead, loss of PGs results in increased rRNA transcription and overall protein translation. PGs modulate these nucleolar functions by tightly regulating nuclear actin, which is enriched in the nucleolus. Specifically, we find that loss of PGs results in both increased nucleolar actin and changes in its form. Increasing nuclear actin, by either genetic loss of PG signaling or overexpression of nuclear targeted actin (NLS-actin), results in a round nucleolar morphology. Further, loss of PGs, overexpression of NLS-actin or loss of Exportin 6, all manipulations that increase nuclear actin levels, results in increased RNAPI-dependent transcription. Together these data reveal PGs carefully balance the level and forms of nuclear actin to control the level of nucleolar activity required for producing fertilization competent oocytes.

## Introduction

Prostaglandins (PGs) are a conserved class of lipid signaling molecules that signal locally near their sites of synthesis (Funk, 2001). PGs are produced by a series of synthases and signal *via* G protein-coupled receptors (GPCRs). Specifically, the fatty acid arachidonic acid serves as the substrate for cyclooxygenase (COX) enzymes, which produce the PG intermediate. PG-type specific synthases convert this intermediate into bioactive PGs which activate PG-type specific GPCRs.

PGs regulate almost every aspect of female reproduction (Tootle, 2013). For example, loss of PG synthesis blocks mammalian follicle maturation and ovulation (Lim et al., 1997; Langenbach et al., 1999; Takahashi et al., 2010). Further, COX inhibitors (non-steroidal anti-inflammatory drugs) cause reversible infertility in women due to similar defects (Akil et al., 1996; Smith et al., 1996; Pall et al., 2001). While it is clear how PGs are produced and initiate signaling cascades, the cellular roles of PGs during follicle development remain unclear.

One poorly understood cellular target of PG signaling is the nucleolus. The nucleolus is the largest nuclear organelle and is best known for its role in ribosomal RNA (rRNA) transcription and ribosome biogenesis (Pederson, 2011; Tiku and Antebi, 2018). It forms by liquid-liquid phase separation (Lafontaine et al., 2021). This phase separation is driven by interactions between intrinsically disordered proteins and rRNA. These interactions are driven by nucleolar function, specifically rRNA transcription. Thus, if the function of the nucleolus is perturbed, its morphology is changed. PG signaling, from flies to humans, regulates nucleolar morphology (Stark et al., 2001; Stark and Dunlop, 2005; Thoms et al., 2007; Khandelwal et al., 2011; Groen et al., 2015), suggesting it controls nucleolar functions. Dynamic regulation of nucleolar activity, including ribosome biogenesis, is a conserved process during oogenesis (Sanchez et al., 2016; Mercer et al., 2021). Specifically, nucleolar function within the oocyte is essential for producing sufficient ribosomes necessary for embryonic development outside of the mother, or for preimplantation embryonic development in placental mammals (Fulka and Aoki, 2016; Kresoja-Rakic and Santoro, 2019; Fulka et al., 2020). As both nucleolar functions and PGs are critical for producing viable oocytes, and PGs regulate nucleolar morphology in other contexts, one cellular role of PG signaling may be to regulate nucleolar function during oogenesis.

To uncover the roles of PGs and their downstream effectors in regulating the nucleolus, we use the robust, *in vivo* genetic system of *Drosophila* oogenesis. Adult female flies have a pair of ovaries, each comprised of ∼15 ovarioles or chains of sequentially developing follicles (also referred to as egg chambers). There are 14 morphologically distinct stages of follicle development. Each follicle is comprised of 16 germline cells—15 nurse cells and one oocyte–which are surrounded by a somatic epithelium of ∼650 follicle cells (Spradling, 1993). We previously identified Pxt as the sole COX-like enzyme in *Drosophila*; thus, loss of Pxt results in a lack of all PG synthesis and signaling (Tootle and Spradling, 2008). Pxt, like mammalian COX enzymes (Tootle, 2013; Prates et al., 2014; Sugimoto et al., 2015), is required for successful follicle development and fertility (Tootle and Spradling, 2008; Tootle et al., 2011). Loss of Pxt results in striking nucleolar morphology changes; the nucleoli in the nurse cells shift from a tubular to a rounded morphology (Groen et al., 2015), strongly suggesting nucleolar function is altered.

PG signaling also plays a conserved and critical role in regulating actin (Peppelenbosch et al., 1993; Pierce et al., 1999; Dormond et al., 2002; Tamma et al., 2003; Bulin et al., 2005; Birukova et al., 2007). Loss of Pxt results in severe defects in actin cytoskeletal remodeling, blocking normal follicle morphogenesis (Tootle and Spradling, 2008; Groen et al., 2012; Spracklen et al., 2014b; Spracklen et al., 2019). In addition to being a key component of the cytoskeleton, actin localizes to, and has numerous conserved functions within the nucleus (Kelpsch and Tootle, 2018; Green et al., 2021). The nuclear localization of actin is highly regulated. Cofilin-actin complexes are imported into the nucleus by Importin 9 (Imp9, (Dopie et al., 2012)), whereas Profilin-actin complexes are exported from the nucleus by Exportin 6 (Exp6, (Stuven et al., 2003)). Additionally, a recent study suggests that in *Drosophila* there are multiple mechanisms controlling nuclear actin import and export to ensure levels are tightly regulated (Borkuti et al., 2022). Inside the nucleus, actin promotes general transcription, is an active component of multiple chromatin remodeling complexes, participates in DNA damage repair, and contributes to nuclear structure (Kelpsch and Tootle, 2018; Green et al., 2021). Given this wide range of nuclear activities, it is not surprising that nuclear actin is emerging as a key regulator of differentiation and cell fate, including during oogenesis (Sen et al., 2015; Misu et al., 2017; Sen et al., 2017; Duan et al., 2020). Further, nuclear actin regulates chromatin movement during meiosis (Mogessie and Schuh, 2017), and plays critical roles in the structure and organization of large oocyte nuclei (Bohnsack et al., 2006); indeed perturbing nuclear actin causes chromatin and nucleolar coalescence (Maslova and Krasikova, 2012; Feric and Brangwynne, 2013). While nuclear actin has many functions, how these functions are regulated, and which are important for oocyte development remain poorly understood.

During *Drosophila* oogenesis, we previously found that there are three pools of endogenous nuclear actin that localize to the nucleolus with distinct developmental patterns (Wineland et al., 2018). These findings suggest that nuclear actin likely functions in the nucleolus. Supporting this idea, actin localizes to nucleoli in many organisms (Jockusch et al., 1971; Funaki et al., 1995; Andersen et al., 2002; Scherl et al., 2002; Andersen et al., 2005; Cruz and Moreno Diaz de la Espina, 2009; Belin et al., 2015; Wineland et al., 2018), numerous actin binding proteins are found in the nucleolus (Hubert et al., 2008; Deng et al., 2012; Groen et al., 2015; Kitamura et al., 2015), and actin promotes RNA Polymerase I (RNAPI) activity (Fomproix and Percipalle, 2004; Philimonenko et al., 2004; Almuzzaini et al., 2016). Given the relationships between PGs, actin, and the nucleolus, we hypothesize that PG signaling regulates nuclear actin to control nucleolar function and morphology during oogenesis.

We find PG signaling negatively regulates nuclear actin to restrict nucleolar activity to the correct level during *Drosophila* follicle development. Loss of Pxt, the *Drosophila* COX-like enzyme, causes striking changes in nucleolar morphology, and surprisingly, these nucleoli have increased rRNA transcription and normal nucleolar localization of a key RNAPI regulator. This ultimately results in increased protein translation. Loss of PG signaling also increases the level and/or expands the developmental pattern of the three pools of nuclear actin, raising the possibility that these changes in nuclear actin drive the alterations in nucleolar function and morphology. Supporting this idea, increasing nuclear actin levels by two different means or co-reduction of Pxt and Exp6, an export factor for nuclear actin (Stuven et al., 2003), results in *pxt*-like increases in rRNA transcription. Together these data lead to the model that PG signaling tightly controls nuclear actin–preventing the accumulation of too much of any nuclear actin pool–to limit nucleolar activity to the levels needed for successful *Drosophila* follicle development. We speculate that this pathway likely regulates oocyte development across organisms, as all the components–PGs, nuclear actin, and nucleolar activity–play conserved roles in oogenesis and fertility.

## Results

### Prostaglandin signaling restricts nucleolar transcription and protein synthesis

**FIGURE 1:**
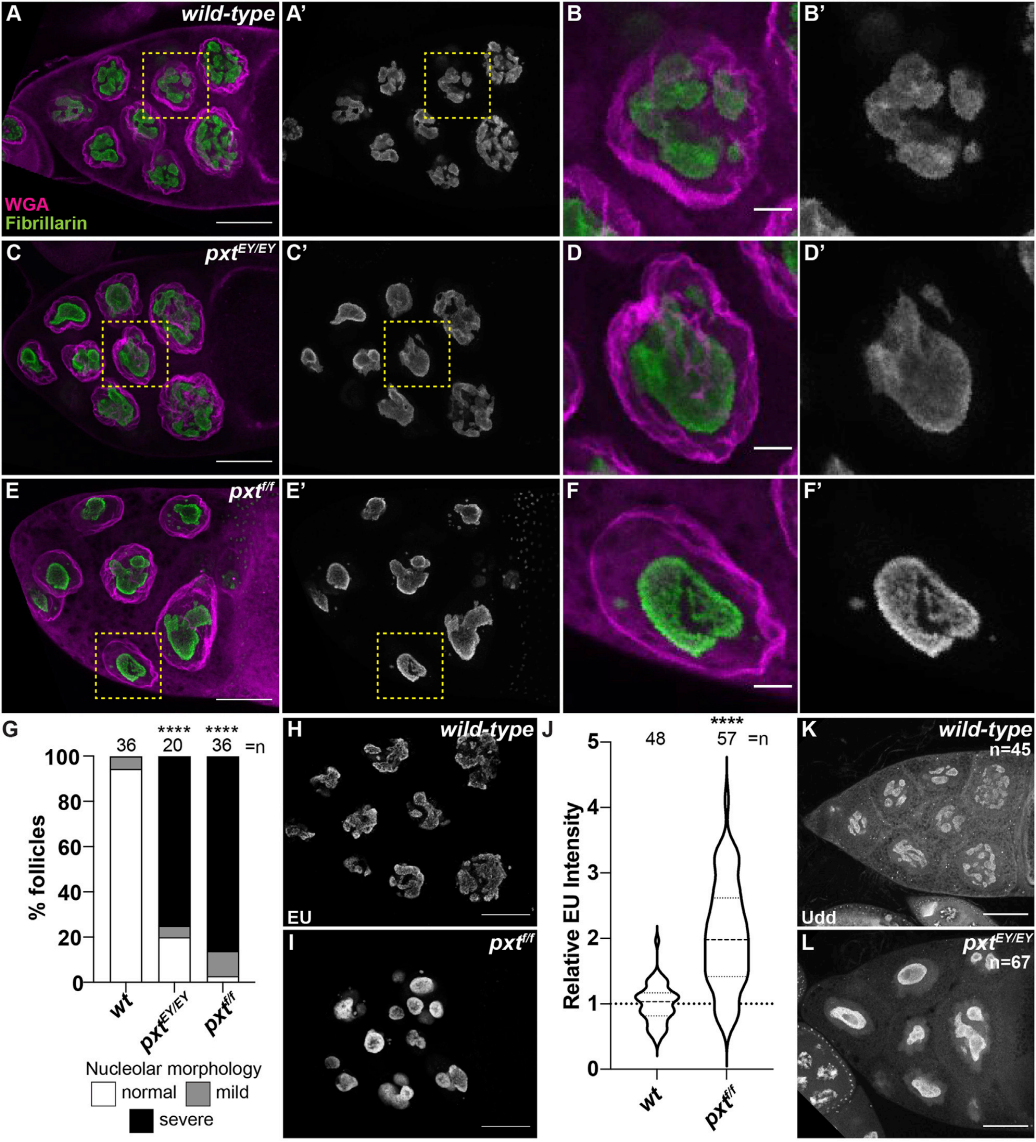
Prostaglandins regulate nucleolar structure and limit rRNA transcription. **(A–F′), (H,I,K,L)**. Maximum projections of 2-4 confocal slices of wild-type (*yw*) and *pxt* mutant (*pxt* ^*EY/EY*^ and *pxt* ^*f/f*^ as indicated) S10B follicles stained for: nucleolus (Fibrillarin, green in merge and white in single channel) and nuclear envelope wheat germ agglutinin (WGA), magenta in merge) in **(A–F′)**, nascent RNA (EU) in **(H,I)**, and Udd, a RNAPI regulatory complex component in **(K,L)**. Yellow boxed regions in **(A, C, E)** are shown as zoomed in images in **(B-B′, D-D′, F-F′)**, respectively. Scale bars = 50 μm except in **(B-B′, D-D′, F-F′)** where scale bars = 10 μm. **(G)**. Graph quantifying the percentage of follicles exhibiting normal (white) vs. mild (gray) or severe (black) defects in nucleolar morphology. **(J)**. Graph of quantification of relative EU intensity. ****p < 0.0001 (unpaired t-test). *n* = number of follicles. Loss of Pxt results in a rounded nucleolar morphology **(A–G)**, increased nascent nucleolar RNA **(H–J)**, and normal nucleolar localization of Udd **(K,L)**.

PGs regulate the structure of the nucleolus, including in *Drosophila* (Stark et al., 2001; Stark and Dunlop, 2005; Thoms et al., 2007; Khandelwal et al., 2011; Groen et al., 2015). In the nurse cells of developing follicles nucleoli appear reticular in early stages (Supplementary Figures 1A-A′, D-D′) and in later stages (from Stage 9 [S9] onward) exhibit an interconnected tubular morphology (Supplementary Figure 1E-E′; Figure 1A-B′ and data not shown). Loss of Pxt by either of two alleles (*pxt* ^*EY*^ and *pxt* ^*f*^)and thereby, loss of all PG synthesis, results in severe changes in nucleolar morphology throughout oogenesis (Supplementary Figure S1 and Figure 1), but the changes are most striking at S10B when almost all nurse cells exhibit rounded nucleoli (Figure 1C-F″) (Groen et al., 2015). Quantification reveals that 80% of *pxt* ^*EY*^ mutant S10B follicles and ∼97% of *pxt* ^*f*^ mutant S10B follicles exhibit nucleolar morphology changes, with the majority of the changes being severe (Figure 1G); the phenotype of the two *pxt* alleles are not statistically different from each other so throughout the study, in most cases, they are used interchangeably and data from the two alleles is often combined and presented as *pxt−/−*. As nucleolar structure is highly dependent on its functions, we sought to identify the changes in nucleolar function that drive the morphological alteration when PGs are lost.

The best understood role of the nucleolus is to transcribe rRNA. Using a nucleotide incorporation assay (Click-iT EU), we assessed nascent RNA production in the nucleolus. In S10B nurse cells nascent RNA is restricted to the nucleolus (Figure 1H), as previously observed (Zhang et al., 2014; Sanchez et al., 2016). Further supporting this approach labels nascent rRNA, inhibition of RNAPII by α-amanitin does not impact the level of nucleolar RNA, whereas inhibition of RNAPI with actinomycin D blocks nucleolar RNA labeling (Supplementary Figures 2A-D) (Zhang et al., 2014; Sanchez et al., 2016). Based on the altered nucleolar morphology in *pxt* mutants, we expected impaired rRNA transcription. Instead, our data shows that loss of Pxt results in increased EU intensity (Figures 1H–J). This finding could indicate an increase in RNAPI activity and rRNA production or it could be an artifact due to the altered nucleolar volume due to the morphology change. To address if nucleolar volume impacts EU intensity we took advantage of the fact that within a wild-type follicle the anterior nurse cell, and therefore its nucleolus, is significantly smaller than more posterior nurse cells. Indeed, the mean nucleolar volume of the anterior-most wild-type nurse cell is 4,211 μm^3^, whereas in the posterior nurse cells it is 5,340 μm^3^ (*p* = 0.0098, paired t-test). We compared EU intensity between these two populations of nurse cells and find that it is similar, indicating that EU intensity and nascent rRNA production is consistent within a follicle, reflecting its stage in development and not the specific nucleolar volume of individual nurse cells (Supplementary Figure S3). These data lead us to favor the model that loss of Pxt results in increased rRNA production. Further supporting that the rounded nucleolar morphology in *pxt* mutants is not due to impaired rRNA production, directly inhibiting RNAPI results in distinct nucleolar morphology changes. Specifically, treatment of wild-type S10B follicles with actinomycin D results in the formation of multiple small, round nucleoli per cell (Supplementary Figure 2E, F). Finally, Udd, a component of the RNAPI regulatory complex (Zhang et al., 2014), remains highly enriched in the nucleoli when Pxt is lost (Figure 1K,L). These findings support the model that loss of Pxt results in increased RNAPI activity.

**FIGURE 2:**
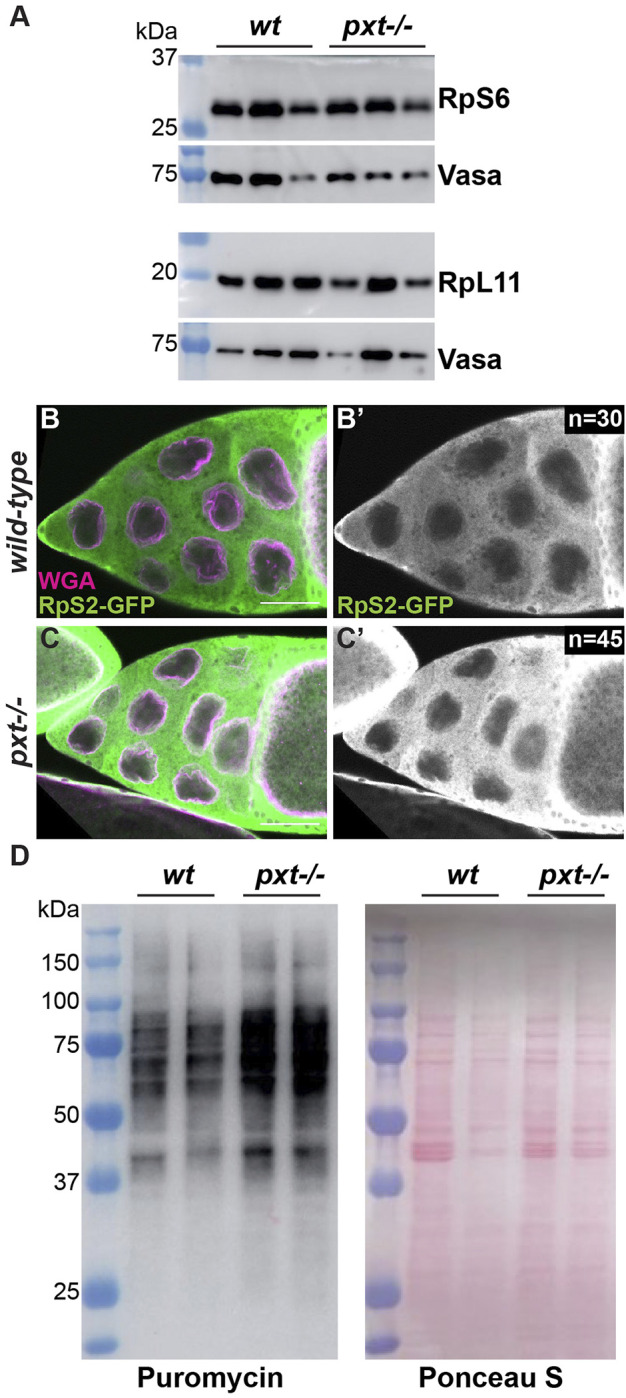
Prostaglandins restrict protein translation levels. **(A)**. Western blots of wild-type and *pxt* ^*EY*^ mutant whole ovary lysates for RpS6 and RpL11; the Vasa blots serve as the loading controls. For RpS6, the blot was cut horizontally at the 37 kDa ladder band, with the top stained for Vasa and the bottom for RpS6. For RpL11, two gels were loaded with the same samples. **(B-C’)**. Single confocal slice of wild-type and *pxt* ^*f*^ mutant S10B follicles stained for: RpS2-GFP (anti-GFP) in green in merge and white in single channel **(B′,C′)** and WGA, which marks the nuclear envelope, in magenta in merge. Scale bars = 50 μm. **(D)**. Representative western blot for Puromycin and Ponceau S stained blot (loading control) of puromycin incorporation assay on wild-type and *pxt* ^*EY*^ mutant whole ovary samples. In **(A,D)**, molecular weight ladder (kDa) is BioRad Precision Plus Protein Standard. Loss of Pxt results in no change in RpS6 compared to RpL11 levels **(A)**, normal cytoplasmic localization of RpS2 **(B-C′)**, and increased protein translation **(D)**.

Our findings in *Drosophila* are consistent with what has been observed in colon cancer cells upon aspirin treatment, which inhibits COX activity and thus PG synthesis (Brighenti et al., 2016). Aspirin treatment results in a reduction of the small ribosomal subunit protein RpS6 compared to the large subunit protein RpL11. This imbalance in ribosomal proteins leads to the buildup of an rRNA intermediate (Brighenti et al., 2016). To determine if the increased nascent nucleolar RNA we observe in *pxt* mutants is due to the same mechanism we assessed RpS6 and RpL11 levels. We find RpS6 remains highly expressed when Pxt is lost (Figure 2A), suggesting loss of Pxt during *Drosophila* oogenesis results in nucleolar changes distinct from those in cancer cells due to aspirin treatment.

We next assessed the role of PGs in ribosomal biogenesis and activity. If ribosome formation is impaired, ribosomal proteins are retained in the nucleolus (Zhang et al., 2014). Using a GFP protein trap in the ribosomal protein RpS2, we find that RpS2 leaves the nucleolus and localizes to the cytoplasm in both wild-type and *pxt* mutant follicles (Figure 2B-C’). This finding suggests that there are no defects in ribosomal biogenesis when Pxt is lost. We then assessed ribosomal activity by visualizing protein translation levels using a puromycin incorporation assay (Schmidt et al., 2009; Jang et al., 2021). Puromycin is a tRNA analog that is incorporated into polypeptide chains during translation. This incorporation results in the premature termination of translation and a puromycin-labeled polypeptide. These puromycin-labeled polypeptide chains can be detected *via* western blot using a puromycin antibody, revealing the relative rates of translation (Aviner, 2020). Loss of Pxt results in increased puromycin incorporation (Figure 2D), suggesting an increased rate of protein synthesis. Together, these findings support the model that PGs normally restrict RNAPI activity, ribosome production, and protein translation during *Drosophila* oogenesis.

### Prostaglandins regulate nuclear actin accumulation in the nucleolus and its polymerization state

We previously discovered that during *Drosophila* oogenesis, PG signaling regulates actin cytoskeletal remodeling, (Tootle and Spradling, 2008; Groen et al., 2012; Spracklen et al., 2014b), and nuclear actin is both dynamic and enriched in the nucleolus (Wineland et al., 2018). These findings led us to hypothesize that PG signaling regulates nuclear actin to modulate nucleolar activities.

**FIGURE 3:**
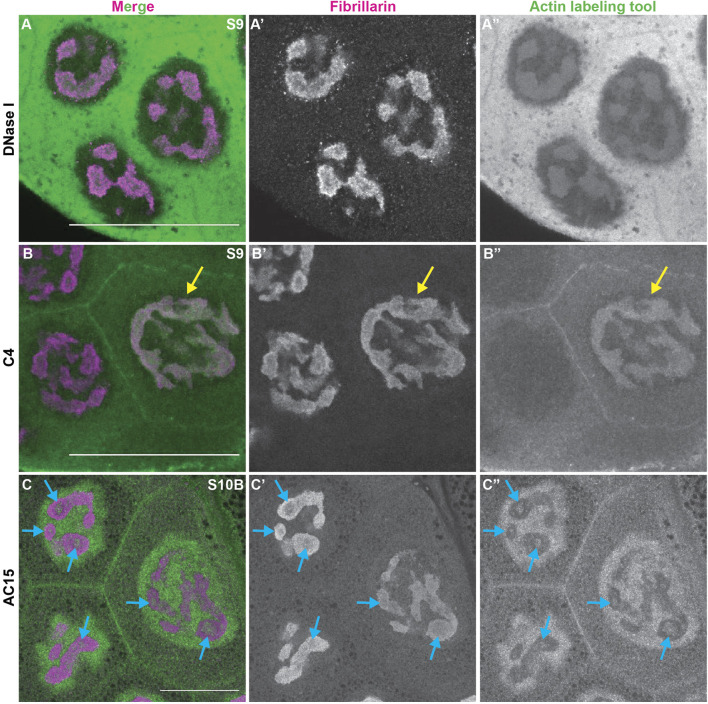
Nuclear actin is enriched in the nucleolus. **(A-C”)**. Maximum projections of 2-5 confocal slices of zoomed in regions of a few nurse cells of wild-type follicles of the indicated stages (S) stained for the nucleolus (Fibrillarin, magenta in merge) and three different nuclear actin labeling tools (green in merge): DNase I **(A-A”)**, anti-actin C4 (C4, **(B-B”)**), and anti-actin AC15 (AC15, **(C-C”)**); scale bars = 50 μm. Yellow arrow indicates a C4 positive nucleolus and blue arrows indicate AC15 positive nucleolar puncta. DNase I, which labels all monomeric actin, is enriched throughout the nucleolus of every cell **(A-A”)**), whereas C4 labels whole nucleoli of a subset of nurse cells (yellow arrow, (B-B”)). AC15 nuclear actin is largely localized to the chromatin, but in mid-oogenesis it labels puncta within every nurse cell nucleoli **(C-C”)**, blue arrows).

During *Drosophila* oogenesis there are three pools of endogenous nuclear actin recognized by DNase I, anti-actin C4 and anti-actin AC15, and all three pools exhibit nucleolar localization (Wineland et al., 2018). DNase I tightly binds to monomeric or G-actin (Hitchcock, 1980). We find that in addition to labeling G-actin in the cytoplasm, in the nucleus DNase I is enriched throughout the nucleolus and colocalizes with Fibrillarin in every single cell during oogenesis (Figure 3A-A″, Supplementary Figure 4A-B″). This finding indicates monomeric nuclear actin is enriched in the nucleolus (Wineland et al., 2018). Like DNase I, anti-actin C4 labels G-actin in both the cytoplasm and nucleus during oogenesis. However, the C4 antibody colocalizes with Fibrillarin, and thus, labels the whole nucleolus, in only a subset of mitotic follicle cells and in some of the nurse cells during S5-9 (Figure 3B-B″ yellow arrow, Supplementary Figure S4C-D″); in these instances, it overlaps with DNase I staining (Wineland et al., 2018). It is important to note that while in any fixed image only some of the nurse cell nucleoli are C4 positive, but based on the pattern of the staining being either middle vs. anterior and posterior nurse cells, we suspect that every nurse cell nucleolus at some point (and likely multiple points) in development has C4 positivity. C4 also labels polymeric actin in undifferentiated germ cells and in the germinal vesicle (Kelpsch et al., 2016; Wineland et al., 2018). We have previously validated the specificity of the C4 antibody for immunofluorescence during *Drosophila* oogenesis (Kelpsch et al., 2016). We next assessed anti-actin AC15 (see Supplementary Figure S5 for validation of AC15 antibody specificity). We find that anti-actin AC15 labels polymeric nuclear actin in every nurse and follicle cell starting weakly around S6, with higher levels in the nurse cells (Wineland et al., 2018). The AC15 levels increase in both the nurse and follicle cells during the subsequent stages with maximal labeling at S10 (Wineland et al., 2018). While AC15 nuclear actin primarily localizes to the chromatin, during mid-oogenesis AC15 nuclear actin puncta are also observed within the nurse cells in regions lacking DNA (Wineland et al., 2018). Previously, we speculated that these AC15 puncta were within the nucleolus (Wineland et al., 2018). To test this, we co-labeled follicles for the nucleolar protein Fibrillarin and AC15. Indeed, we find that AC15 labels puncta within the nurse cell nucleoli during S9-12 (Figure 3C-C″ blue arrows, Supplementary Movie S1, Supplementary Figure S4E-E” and data not shown). Together, these findings reveal that all three pools of endogenous nuclear actin localize to the nucleolus during follicle development, suggesting nuclear actin functions within the nucleolus.

**FIGURE 4:**
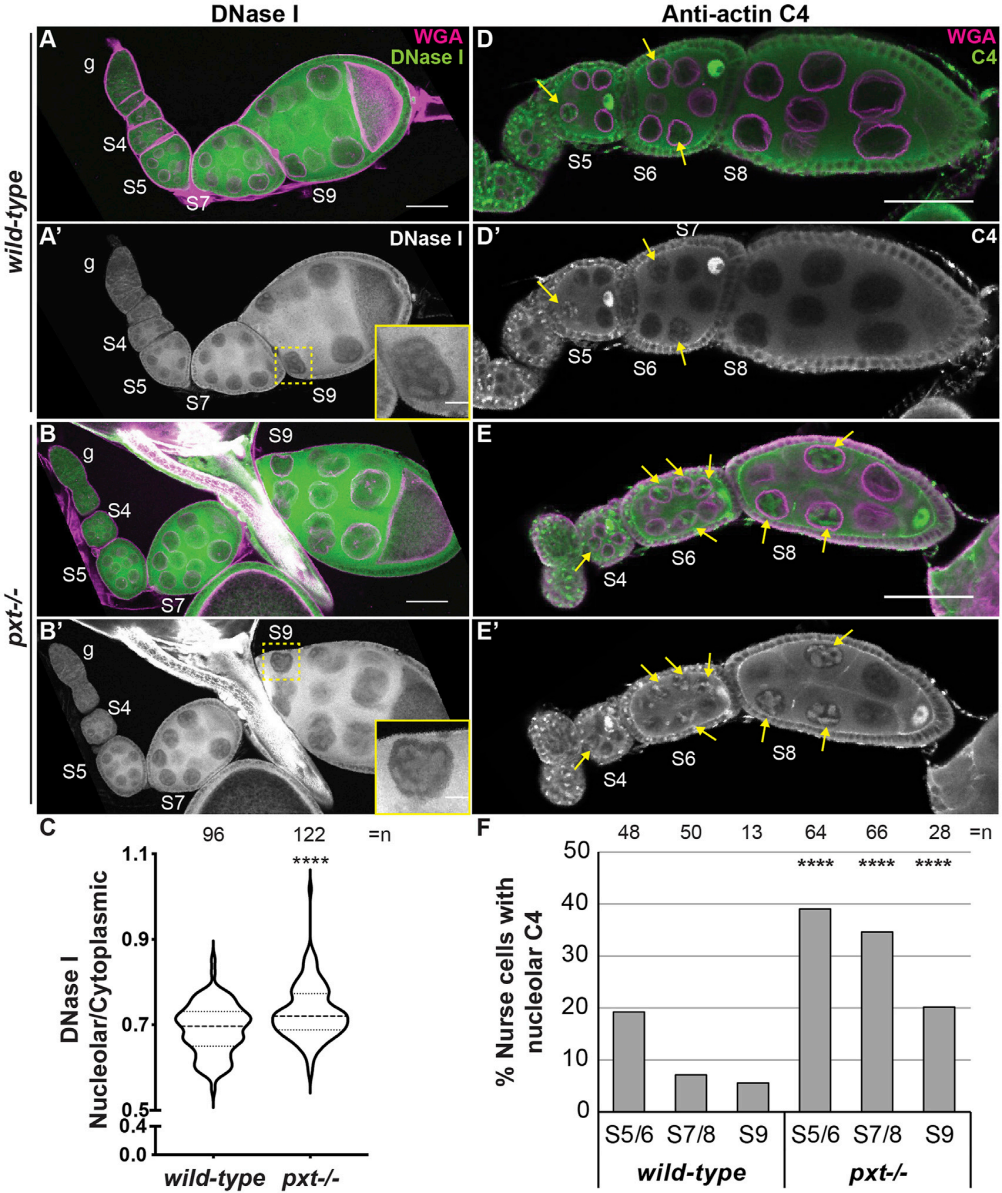
Prostaglandins restrict the amount of nuclear G-actin. **(A-B′)**, **(D-E’)**. Maximum projections of two confocal slices of wild-type and *pxt* ^*EY*^ mutant follicles of the indicated stages (S, germarium = g) stained for: nuclear envelope (WGA) in magenta in merge and either DNase I **(A-B′)** or anti-actin C4 (C4, **(D-E′)** in green in merge and white in single channel. In **(A′,B′)**, yellow dashed box indicates nurse cell in inset. In **(D-E′)**, yellow arrows indicate C4 positive nurse cell nucleoli. Scale bars = 50μm, except in inset where scale bars = 10 μm. **(C)**. Graph quantifying the ratio of nucleolar to cytoplasmic DNase I fluorescence intensity in S7/8 follicles, ****p < 0.0001 (unpaired t-test with Welch’s correction). n = number of nurse cells; three nurse cells were measured per follicle. **(D)**. Graph quantifying the percentage of nurse cells in S5/6, S7/8, and S9 with C4 nucleolar staining, ****p < 0.0001 (Pearson’s Chi-squared test). *n* = number of follicles. In **(C,D)**, data from both *pxt* alleles were combined. Loss of Pxt results in increased nucleolar DNase I levels **(A–C)**, and an increased frequency of C4 positive nurse cell nucleoli **(D–F)**.

We next asked whether PGs regulate the three pools of nuclear actin. Loss of Pxt results in increased nucleolar G-actin, as seen by DNase I and C4 labeling (Figure 4). We performed DNase I staining of *wild-type* and *pxt* mutant ovaries in the same tube; co-labeling with the Pxt antibody to distinguish the genotypes. Loss of Pxt results in subtle but significant increase in nucleolar DNase I staining in the nurse cells (Figure 4B-B′ compared to Figure 4A-A’), and a higher nucleolar to cytoplasmic DNase I fluorescence intensity ratio compared to *wild-type* (Figure 4C). We next examined the role of PGs in regulating C4 nuclear actin. We did not observe differences between C4 labeling in wild-type and *pxt* mutant follicle cells (data not shown). However, loss of Pxt results an increased frequency of nurse cells exhibiting nucleolar C4 staining (Figures 4E, F). These data indicate that loss of Pxt results in a change in the nucleolar G-actin that leads to increased C4 labeling.

**FIGURE 5:**
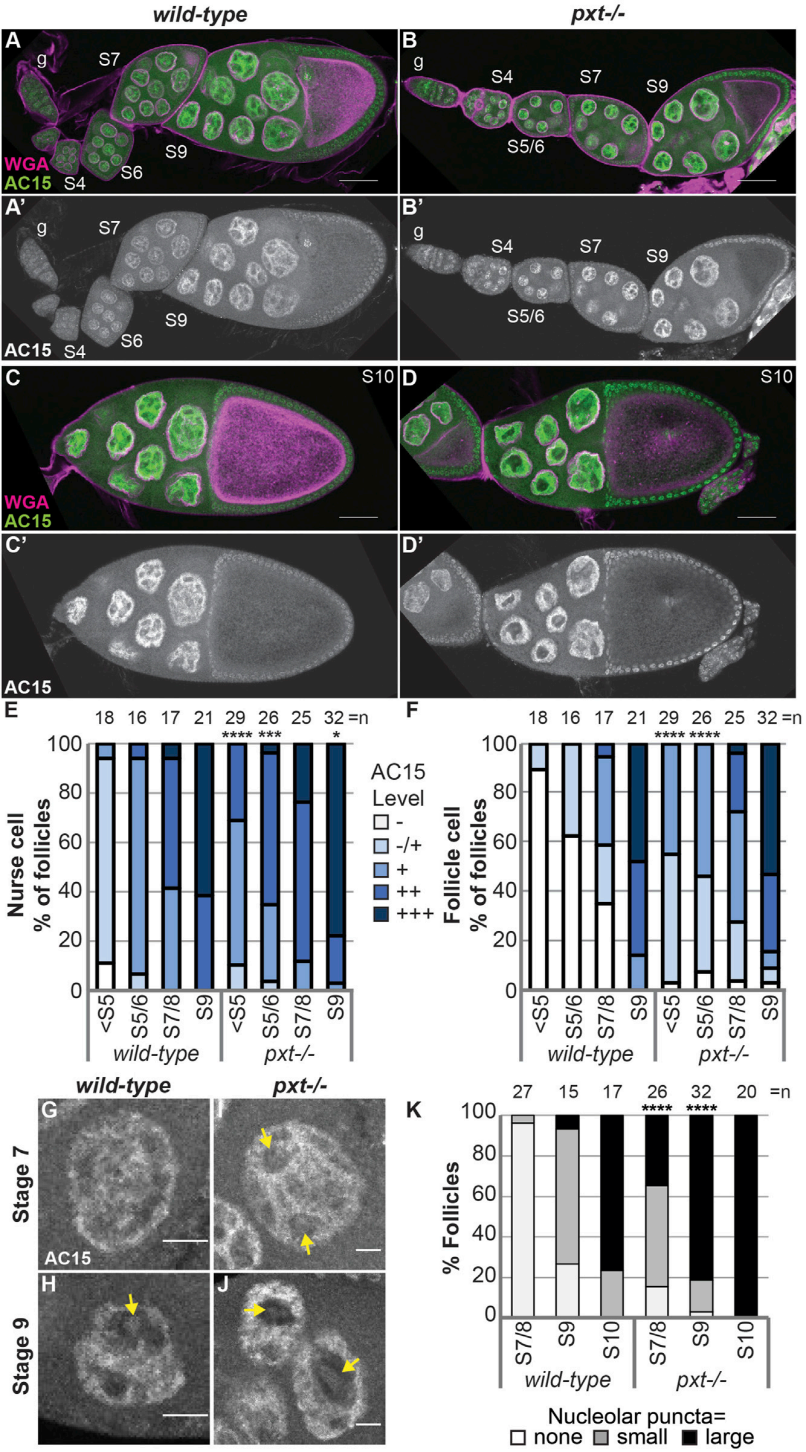
Prostaglandins restrict the developmental pattern and level of AC15 nuclear actin. **(A-D’)**. Maximum projections of two confocal slices of wild-type and *pxt* ^*f*^ mutant follicles of the indicated stages (S, germarium = g) stained for: nuclear envelope (WGA) in magenta in merge and AC15 in green in merge and white in single channel; scale bars = 50 μm. **(E,F)**. Graphs quantifying the percentage of follicles at the indicated stages with different levels of AC15 (−, −/+, +, ++, +++, white to dark blue) staining for nurse cells **(E)** and follicle cells **(F)**, ****p < 0.0001, ***p < 0.001, **p < 0.01 (Fisher’s exact test). n = number of follicles. **(G–J)**. Maximum projections of two confocal slices of wild-type and *pxt* ^*f*^ mutant nurse cells of the indicated stages stained for anti-actin AC15 (AC15) from the same images in **(A′,B’)**. Yellow arrows indicate puncta. Scale bars = 10 μm. **(K)**. Quantification of the percentage of follicles of the indicated stages exhibiting no (white), small (gray) or large (black) nucleolar puncta, ****p < 0.0001 (Pearson’s Chi-squared test). *n* = number of follicles. In **(E, F, K)**, data from both *pxt* alleles were combined. Loss of Pxt results in earlier and stronger nuclear AC15 staining in both the nurse and follicle cells **(A–F)**, and earlier, larger, and more nucleolar AC15 puncta **(G–K)**.

Having found that PGs play a critical role in regulating nuclear G-actin, we assessed if PGs similarly affects polymeric nuclear actin recognized by AC15. Loss of Pxt results in earlier and more uniform AC15 labeling during *Drosophila* oogenesis (Figure 5A-D’); the *wild-type* and *pxt* mutant follicles were stained in the same tube, indicating the difference is not due to a staining artifact. We quantified the AC15 level in different developmental stages on a five-point scale (see methods for details). Loss of Pxt results in increased nuclear AC15 labeling prior to S5 and in S5/6 nurse and follicle cells, while the later stages are similar to wild-type (Figures 5E, F). These data suggest that loss of Pxt increases the amount of polymeric nuclear actin in the earlier stages of oogenesis, suggesting PGs regulate the form of nuclear actin. Given that AC15 labels puncta within nucleoli during mid-oogenesis (Figure 3C-C″ blue arrows, Movie 1, and Supplementary Figure S4E-E”), we next examined whether PGs regulate AC15 nucleolar actin. In wild-type follicles, there are little to no AC15 nucleolar puncta during S7/8, small puncta are observed in the majority of S9 follicles, and by S10, all follicles exhibit puncta and most puncta are large (Figures 5G, H, K). Loss of Pxt results in earlier and increased AC15 nucleolar puncta, and these puncta are larger than those in wild-type follicles (Figures 5I–K).

Together our studies reveal that PGs play an important role in limiting the level of nucleolar actin and regulating its form–G-actin vs. C4 G-actin and monomeric vs. polymeric actin (AC15). We speculate that increased nucleolar actin affects both the stage it is observed in and later stages due to downstream effects of the impacted processes. Indeed, the nucleolar morphology defects observed in *pxt* mutants are more striking in the later stages (Kelpsch et al., 2016).

### Increased nuclear actin levels alter nucleolar activity

We next sought to determine if the PG-dependent modulation of nucleolar morphology and function is due to regulation of nuclear actin. If this is the case, then increasing nuclear actin levels alone will drive *pxt*-like changes in nucleolar morphology and/or function. To increase nuclear actin levels, we took two approaches, overexpression of nuclear targeted actin and loss of a nuclear actin export factor, Exp6 (encoded by *Drosophila ellipsoid body open* or *ebo*; (Stuven et al., 2003)).

**FIGURE 6:**
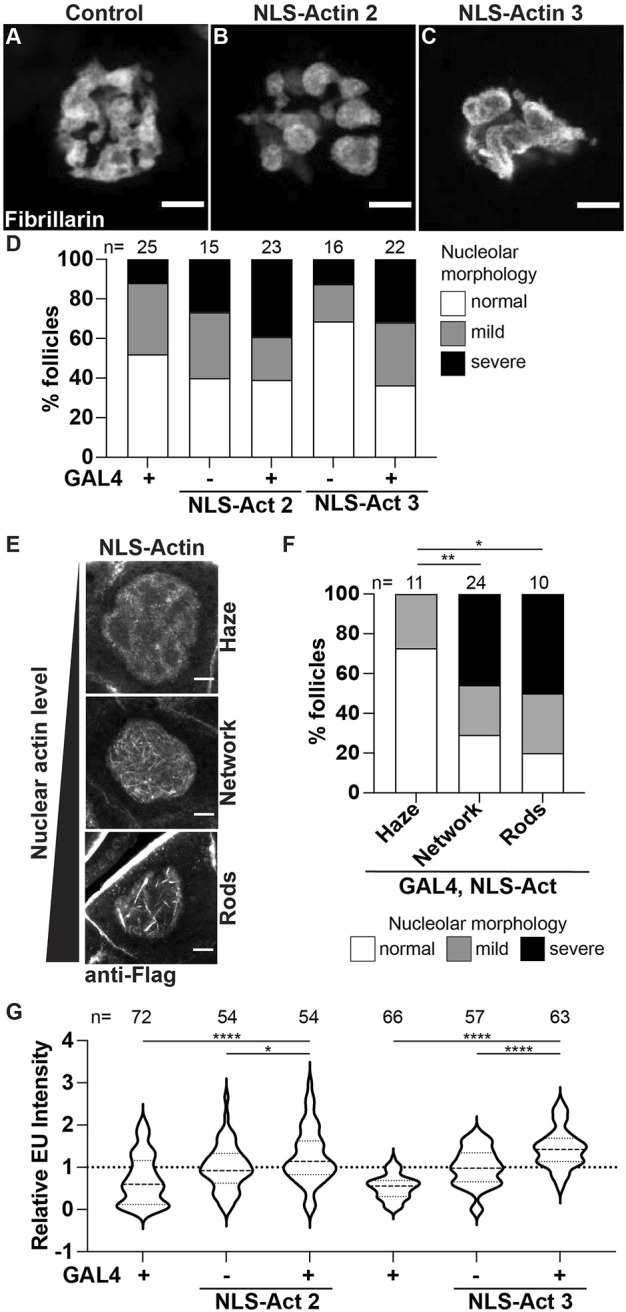
Increasing nuclear actin levels alters nucleolar morphology and increases nucleolar activity. **(A–C)**. Maximum projections of 2-4 confocal slices of Control (*osk GAL4/+*) and NLS-Actin (*osk GAL4/+; UAS NLS-flag-Actin 5C/+*) overexpressing single S10B nurse cells stained for the nucleolus (Fibrillarin). **(D)**. Graph quantifying the percentage of follicles exhibiting normal (white) vs. mild (gray) or severe (black) defects in nucleolar morphology. **(E)**. Maximum projections of 2-3 confocal slices of NLS-Actin overexpressing S10B nurse cells stained for Flag that exhibit varying levels of nuclear actin: haze (no obvious filaments), network of thin filaments, and rods (thick filaments). **(F)**. Graph quantifying the percentage of follicles exhibiting normal (white) vs. mild (gray) or severe (black) defects in nucleolar morphology when all the NLS-Actin overexpressing follicle data was combined and binned based on the level/structure of nuclear actin observed: haze, network of filaments, or rods **p < 0.01, *p < 0.05 (unpaired t-test). n = number of follicles. **(G)**. Graph quantifying the relative EU fluorescence intensity of the indicated genotypes, ****p < 0.0001, *p < 0.05 (unpaired t-test). *n* = number of follicles. Overexpression of NLS-tagged Actin results in variable alterations in nucleolar morphology, including pebbling **(B)** and coalescence **(C)**; these changes are not statistically significant **(D)**. However, when the level of nuclear actin is considered, follicles with increased nuclear actin, as observed by filament networks and rods, nucleolar morphology is altered **(E,F)**. Further, nascent nucleolar RNA levels are increased when nuclear targeted actin is expressed **(G)**. Scale bars = 10 μm.

First, we used the UAS/GAL4 system to overexpress nuclear targeted actin (UASp NLS-flag-Actin 5C, referred to as NLS-Actin) in the germline using two different transgenic insertions. Increasing nuclear actin levels results in altered nucleolar morphology (Figures 6A-C). The nucleoli go from a tubular to a pebbled morphology, and in a few cases trended towards a *pxt*-like rounded phenotype. Notably, these nucleolar changes are only observed in a subset of the nurse cells; this is in contrast to *pxt* mutants in which all the nurse cell nucleoli within a follicle are affected. Quantification of the penetrance of the morphological changes across the nurse cells within a follicle reveals that while increased morphology changes are observed when NLS-Actin is overexpressed, but these changes are not significantly different than the controls (Figure 6D). To determine if this subtle change in nucleolar morphology was due to the variable level of nuclear actin resulting from NLS-Actin expression, we combined the NLS-Actin overexpression data and binned it based on the level of nuclear actin. As expected from previous work (Spracklen et al., 2014a; Kelpsch et al., 2016), when exogenous nuclear actin levels are low, only a nuclear actin haze is observed, whereas increasing nuclear actin levels results in a nuclear actin filament network (many thin actin filaments) or nuclear actin rod formation (fewer, thick actin structures) in the nurse cells (Figure 6E). We find that follicles with apparent nuclear F-actin structures have nucleolar morphology changes (i.e. the presence of nuclear actin filament networks and rods correlates with severe changes in nucleolar morphology), supporting that increased nuclear actin and/or changes in nuclear actin structure cause nucleolar alterations (Figure 6F). We next assessed if these changes in nucleolar structure reflect *pxt*-like increases in nucleolar activity. Overexpression of NLS-Actin results in increased nascent nucleolar RNA (Figure 6G).

We then assessed how loss of Exp6 impacts nucleolar morphology and function. Loss of Exp6 results in mild and variable alterations in nucleolar morphology, with a few follicles exhibiting nurse cells with pebbled nucleoli (Supplementary Figure S6A-C’); notably, these morphological changes are not statistically significant. This lack of phenotype could be due to the nature of the *exp6* allele (*exp6* ^*f*^ *)*. To test this possibility, we assessed Exp6 levels in this and other alleles; we find that *exp6* ^*f*^ is a strong hypomorph with little to no protein present (Supplementary Figure S7A). Thus, the allele is not the reason for the lack of nucleolar morphology change. Instead, we speculate it is due to only minor alterations in nuclear actin levels. Indeed, it has been shown that loss of Exp6 only increases total nuclear actin levels by ∼20% (Borkuti et al., 2022). We next assessed nucleolar function and find loss of Exp6 results in a significant increase in nascent nucleolar RNA (Supplementary Figure S6D). Together these data reveal that tight regulation of nuclear actin is critical for maintaining normal nucleolar activity, and when significantly perturbed impacts nucleolar morphology.

### PGs regulate nuclear actin to control nucleolar functions

**FIGURE 7:**
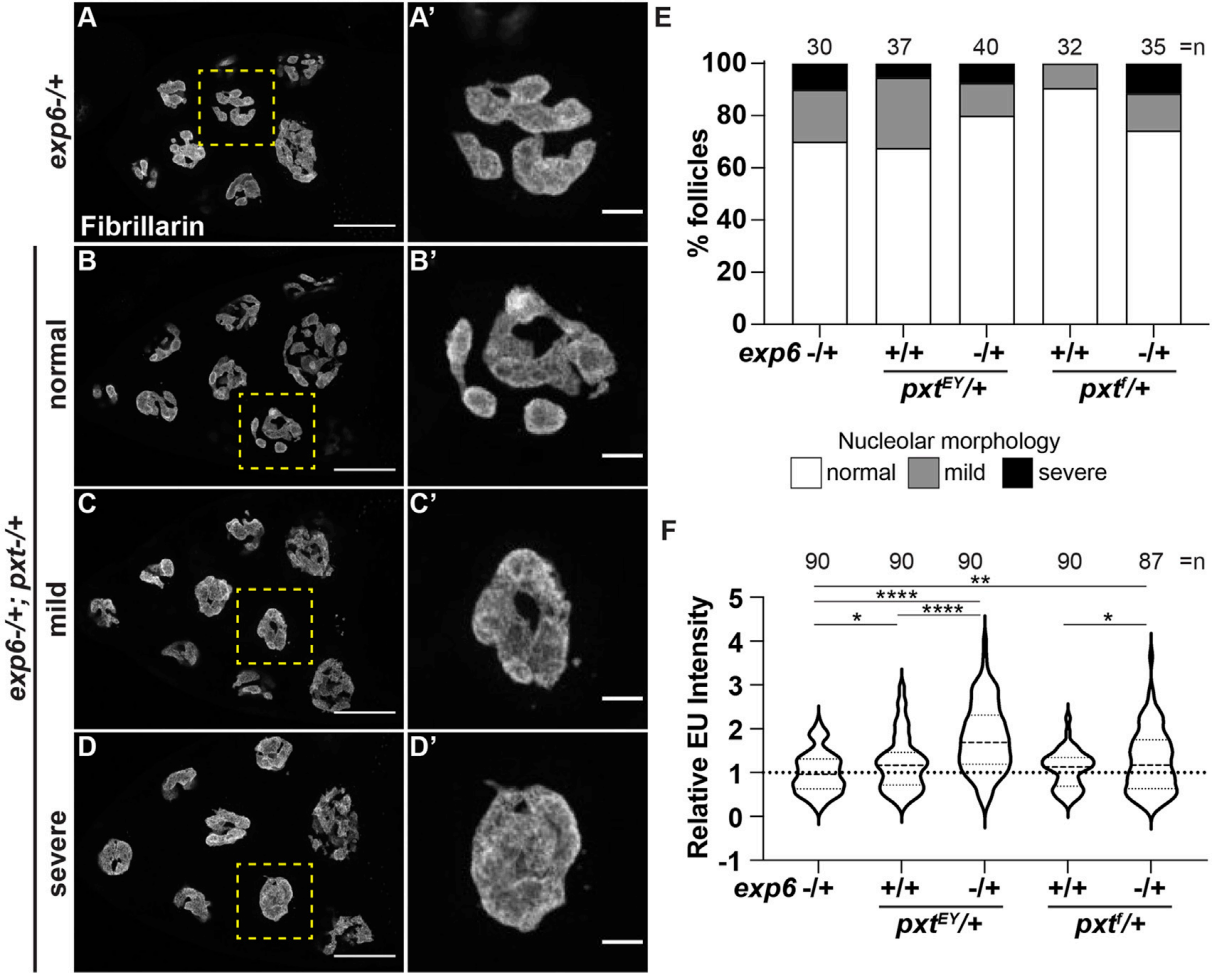
Prostaglandins limit nuclear actin to control nucleolar activity. **(A-D’)**. Maximum projections of 3-4 confocal slices of *exp6−/+* (control) and *exp6−/+; pxt* ^*EY*^ */+* S10B follicles **(A–D)**, scale bars = 50 μm, or zoomed in images of single nurse cells boxed in yellow **(A′-D′)**, scale bars = 10 μm, stained for the nucleolus (Fibrillarin). Examples of the varying nucleolar morphologies observed in the double heterozygotes are shown, normal vs. mild or severe defects. **(E)**. Graph quantifying the percentage of follicles exhibiting normal (white) vs. mild (gray) or severe (black) defects in nucleolar morphology; n = number of follicles. **(F)**. Graph quantifying the relative EU fluorescence intensity of the indicated genotypes, ****p < 0.0001, **p < 0.01, *p < 0.05 (unpaired t-test). n = number of follicles. Heterozygosity for *exp6* has little effect on nucleolar structure **(A-A′)**, whereas follicles from double heterozygotes of *exp6* and *pxt* exhibit varying nucleolar morphologies, from normal to severe defects **(B-D′)**; quantification of the level of defects indicates these changes are not statistically significant **(E)**. In contrast, double heterozygotes of *exp6* and *pxt* exhibit increased nascent nucleolar RNA **(F)**.

**FIGURE 8:**
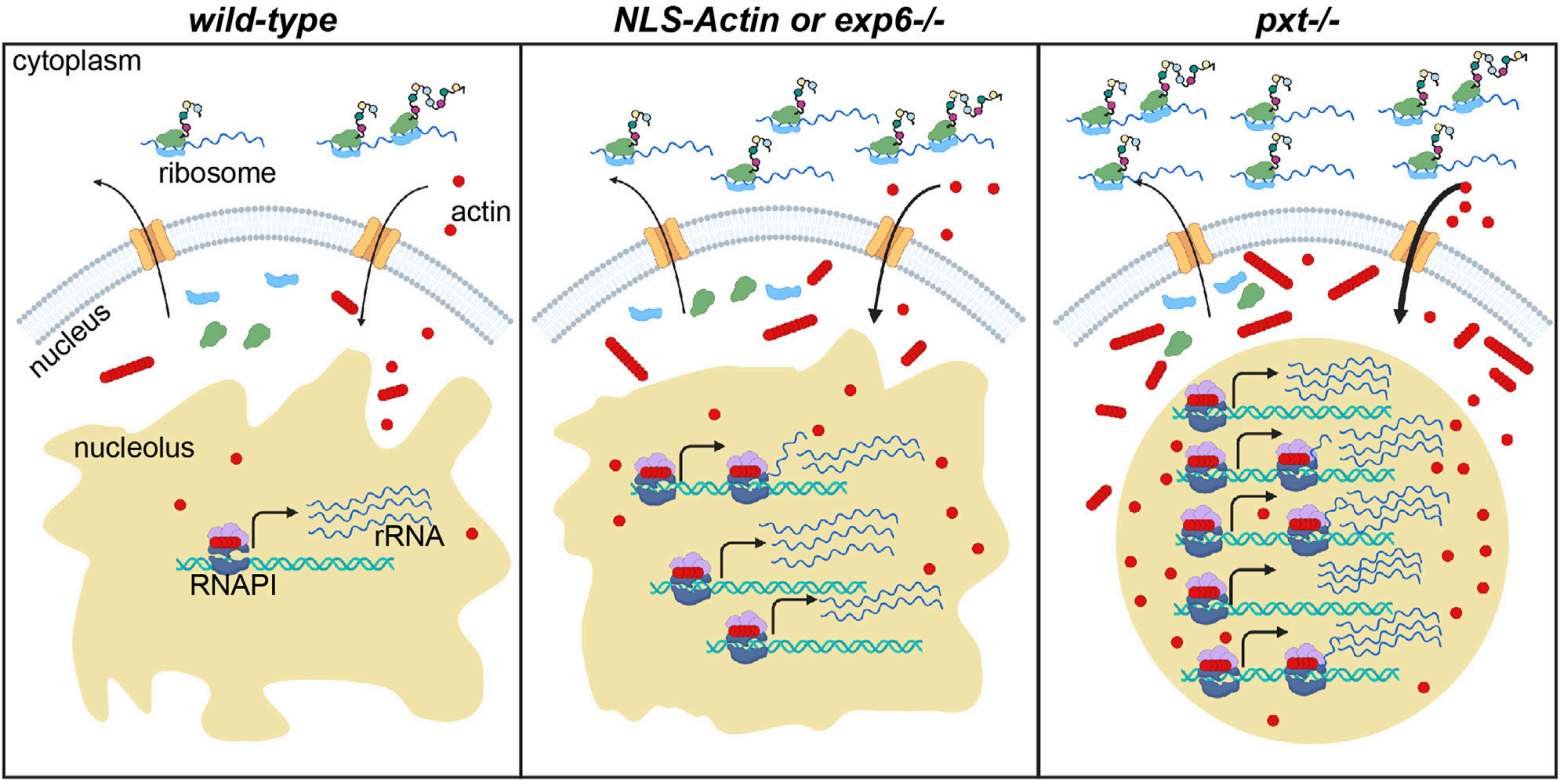
Nuclear actin levels must be tightly regulated to coordinate nucleolar activity with development. Schematic of the resulting model for how nuclear actin levels and/or form correspond to the level of rRNA transcription and protein translation in wild-type, increased nuclear actin (NLS-Actin overexpression or Exportin 6 [Exp6] loss), and *pxt* mutant contexts. The cytoplasm (top) and nucleus (bottom) are diagramed for a single cell in each context. Nuclear envelope = gray, nuclear pores = orange; nucleolus = tan; RNAPI complex: polymerase = dark blue, regulatory factors = light purple, ribosomal DNA (rDNA) = teal helix, rRNA = blue lines; actin (monomers and polymers) = red circles and lines of red circles; ribosomal subunits = light green and light blue; nascent proteins = colored circles on black lines emerging from ribosomes. In wild-type follicles, tight regulation of nuclear actin maintains the correct levels of monomeric and polymeric nucleolar actin to ensure correct rRNA production, a tubular nucleolar structure, and normal ribosome biogenesis and protein translation levels. When nuclear actin levels are slightly increased, by expression of NLS-Actin or loss of Exp6, there is increased nuclear and nucleolar actin, a modest change in nucleolar structure, and increased rRNA production. When Pxt is lost, nuclear actin levels increase and the form of nuclear actin is altered; this results in increased rRNA production, a rounding of the nucleolus, increased ribosome biogenesis (predicted but not tested) and increased protein translation levels.

While our data reveal that PGs and nuclear actin both regulate nucleolar function/morphology and PGs control nuclear actin levels, it remains possible that PGs and nuclear actin act independently to regulate the nucleolus. To test if the nuclear actin changes in *pxt* mutants drive the observed changes in nucleolar activity, we assessed dominant genetic interactions between Pxt and Exp6. Heterozygosity for mutations in either *pxt* or *exp6* should weakly increase nuclear actin levels and therefore, have little to no effect on nucleolar morphology or function. If increasing nuclear actin drives nucleolar changes, then simultaneously reducing these two factors that normally limit nuclear actin levels is expected to increase nuclear actin and cause nucleolar defects. We find that there are variable nucleolar morphology changes in the double heterozygotes (*exp6−/+;;pxt−/+*), ranging from normal to severe *pxt-*like rounded nucleoli (Figure 7A-D’), but these changes are not statistically significant (Figure 7E). However, the double heterozygotes exhibit increased nascent nucleolar RNA levels (Figure 7F). This genetic interaction could suggest that PGs regulate Exp6; however, we think this is unlikely, as the phenotypes of the single heterozygotes are not statistically different from wild-type follicles (*yw*, *n* = 90), and as the most established means of regulating Exp6 activity is by modulating its expression (Bohnsack et al., 2006; Fiore et al., 2017) and Exp6 levels are unchanged in *pxt* mutants (Supplementary Figure S7B). Together, these findings lead to the model that PG signaling restricts nuclear actin to control nucleolar activity, specifically rRNA transcription, and modulates nucleolar morphology (Figure 8).

## Discussion

While the systemic effects of PGs are well characterized, such as their role in regulating female fertility and oocyte development (Tootle, 2013), the cellular mechanisms remain unclear. Here we provide the first evidence that PGs and nuclear actin are critical regulators of the nucleolus during egg development. Specifically, we discovered PGs tightly control the level and forms of nuclear actin. This tight control of nuclear actin is necessary to maintain normal nucleolar functions and morphology. Increasing nuclear actin, through genetic loss of PG signaling or overexpression of an NLS-targeted actin, results in a round nucleolar morphology. Moreover, we find that loss of PGs, overexpression of an NLS-target actin, or loss of Exp6, all manipulations that increase nuclear actin levels, results in an increase in RNAPI-dependent transcription. Further, loss of PGs does not inhibit ribosome formation and maturation, but increases global translation, despite the gross changes in nucleolar morphology. We demonstrate that these changes in the nucleolus are a result of Pxt-dependent nuclear actin regulation. Together, these findings lead to the model that during *Drosophila* oogenesis, PGs limit nuclear actin to promote proper nurse cell nucleolar function and morphology. When these factors become disrupted, it leads to changes in nucleolar function and morphology. These disruptions likely contribute to the sterility in *pxt* mutants (Tootle and Spradling, 2008; Tootle et al., 2011). Given the conservation of PGs, nuclear actin, and the nucleolus, it is tempting to speculate these same mechanisms regulate oocyte development and female fertility across organisms.

### PGs control the functions of the nucleolus

The function of the nucleolus is intimately linked to its structure. The nucleolus is non-membrane bound, and forms by liquid-liquid phase separation (Dubois and Boisvert, 2016; Stochaj and Weber, 2020; Lafontaine et al., 2021). Specifically, rDNA transcription allows intrinsically disordered nucleolar proteins, including Fibrillarin, to coalesce and form the discreet compartments of the nucleolus. This connection between function and structure led to the prevailing notion that changes in nucleolar morphology are indicative of dysfunction (Yuan et al., 2005; Boulon et al., 2010; Grummt, 2013). This idea, along with the prior work showing aspirin impairs nucleolar function in cancer cells (Brighenti et al., 2016; Chen et al., 2018), led us to initially hypothesize the rounded nucleolar morphology observed in *pxt* mutants was the result of impaired nucleolar activity. This is not the case. Instead, we find loss of Pxt results in increased rRNA transcription, normal ribosome biogenesis, and increased protein translation (Figures 1, 2).

The increased transcription observed in the rounded nucleoli of *pxt* mutants is not the first instance of enhanced nucleolar function being associated with altered nucleolar morphology. For example, during *Drosophila* oogenesis, loss of RpS5b, a small ribosomal subunit protein paralog, results in a rounded nucleolar morphology, and increased Udd levels, rRNA transcription, and global translation (Jang et al., 2021). Additionally, cancer cells have striking changes in nucleolar morphology, and increased rates of translation and ribosome production; this is linked to disease progression (Orsolic et al., 2016; Lindstrom et al., 2018; Lafita-Navarro and Conacci-Sorrell, 2022). It is thought that increased flux of RNA, proteins, and other molecules important for rRNA transcription, processing, and maturation within the nucleolus results in a more liquid-like nucleolar state, driving the enlarged and rounded structure (Lafontaine et al., 2021). Similarly, the nucleoli of *Xenopus laevis* oocytes are more rounded and liquid-like than other cell types, and this nucleolar structure is essential for oocyte development and maturation (Brangwynne et al., 2011; Feric et al., 2016). Conversely, solid-like nucleoli are thought to reduce the flux of factors into and out of the nucleolus, and thereby, decrease rRNA transcription and processing, ribosomal precursor release, and ribosome biogenesis; this is observed in patients with ribosomopathies (Lafontaine et al., 2021). Given the rounded morphology of the nucleoli and increased nucleolar functions in *pxt* mutants, it is tempting to speculate that the nucleoli have a liquid-like state whereas wild-type cells have more solid-like nucleoli.

Our finding that genetic loss of COX activity during *Drosophila* oogenesis results in increased nucleolar functions is at odds with the known effects of inhibiting COX activity in cancer cells. Aspirin treatment of both hepatocarcinoma and colorectal cancer cells results in decreased numbers but increased size of nucleoli (Brighenti et al., 2016; Chen et al., 2018). Further, in the colorectal cancer study, aspirin decreases RpS6 expression compared to RpL11; this misbalance in ribosomal proteins impairs ribosome maturation. These ribosomal biogenesis defects feed back to the nucleolus, resulting in the accumulation of a rRNA intermediate (Brighenti et al., 2016). Conversely, we find that loss of Pxt does not result in decreased RpS6 or impaired ribosome activity, but instead increases protein translation (Figure 2). This difference could simply be due to aspirin reducing but not eliminating PG signaling. Furthermore, higher eukaryotes have two COX enzymes, COX1 and COX2. COX1 is more sensitive to aspirin inhibition (Vane and Botting, 2003), and COX2 is highly upregulated in cancer (Hashemi Goradel et al., 2019). COX1 and COX2 also have tendencies to couple with distinct downstream synthases, resulting in the production of different bioactive PGs (Ueno et al., 2001; Yuan and Smith, 2015). Thus, aspirin treatment may result in the reduction of specific PGs but not the loss of all PGs, as occurs in the *Drosophila pxt* mutants. Finally, the cellular context may drive the differences observed. Cancer cell nucleoli are less responsive to mechanical signaling than normal cells (Jaecker et al., 2022). Further, cancer cells are mitotic, and nucleolar stress can drive the death of individual cells. In contrast, the nurse cells in the *Drosophila* follicle are post-mitotic, and can only undergo synchronous cell death (i.e. all the nurse cells in a follicle) at specific developmental time points (Jenkins et al., 2013). Future studies are needed to explore the roles of genetic loss vs. pharmacologic inhibition of PG synthesis in diverse contexts to decipher both the common and cell-specific roles of PGs in regulating the nucleolus.

### What are the consequences of increased rRNA transcription and protein translation?

The level of rRNA transcription and protein translation are tightly tuned for each cell-type, differentiation status, and developmental stage (Buszczak et al., 2014; Mercer et al., 2021). During development, cells significantly increase the level of protein translation as they differentiate. For example, the *Drosophila* ovarian germline stem cells (GSCs) exhibit high levels of rRNA transcription but lower levels of protein translation, whereas the differentiating daughter cells decrease rRNA transcription and increase protein translation (Zhang et al., 2014). Misregulation of this developmental change in nucleolar activity and protein translation impairs germ cell differentiation (Zhang et al., 2014; Sanchez et al., 2016). In aged animals, loss of Pxt results in early oogenesis defects that resemble germ cell differentiation mutants (Tootle and Spradling, 2008); perhaps this phenotype is due to alterations in rRNA transcription and protein translation. The nurse cells of developing *Drosophila* follicles also likely require specific levels of nucleolar and ribosomal activities. Supporting this idea, loss of RpS5b increases rRNA transcription and protein translation, and these changes dramatically impair oogenesis and result in sterility (Kong et al., 2019; Jang et al., 2021). Specifically, it causes mid-oogenesis checkpoint arrest, meaning follicle development stops at S8 and these follicles undergo cell death. Loss of Pxt results in increased mid-oogenesis checkpoint death (Spracklen et al., 2014b), raising the possibility that this phenotype is due to its altered nucleolar activity. Increased rRNA transcription and protein translation also consume lots of the cellular energy, and thereby, reduce what is available for other processes. Indeed, in *RpS5b* mutants the distribution and shape of mitochondria within the nurse cells are severely altered, and there is elevated reactive oxygen species (Kong et al., 2019). While it remains unknown whether loss of PGs causes similar changes, other energy dependent processes are impaired. In particular, actin cytoskeletal remodeling consumes large amounts of ATP and is severely disrupted in *pxt* mutants (Tootle and Spradling, 2008; Groen et al., 2012; Spracklen et al., 2014b). Finally, increased protein translation in the context of RpS5b loss alters the translational efficiencies of specific mRNAs. Of particular interest is that multiple cytoskeletal genes exhibit decreases translation efficiency. This raises another possible mechanism whereby loss of PGs results in actin cytoskeletal defects. Future studies are needed to determine how the increased rRNA transcription and protein translation in the *pxt* mutants impacts oogenesis and fertility.

### PGs regulate nuclear actin

While PGs have been widely implicated in regulating cytoplasmic actin dynamics (Peppelenbosch et al., 1993; Pierce et al., 1999; Dormond et al., 2002; Tamma et al., 2003; Bulin et al., 2005; Birukova et al., 2007), including during *Drosophila* oogenesis (Tootle and Spradling, 2008; Groen et al., 2012; Spracklen et al., 2014b; Spracklen et al., 2019), here we provide the first evidence that PGs control nuclear actin levels and form (monomeric vs. polymeric). Indeed, loss of Pxt, the enzyme required for all PG synthesis in *Drosophila*, results in increased G-actin in the nucleolus, as seen by DNase I and C4 staining (Figure 4) and increased polymeric actin (AC15) on the chromatin and puncta in the nucleolus (Figure 5). Further supporting that the form of nuclear actin is critical for regulating the nucleolus, nucleolar morphology is altered when NLS-Actin expression results in filamentous nuclear actin (Figure 6). These data lead to the model that PGs are required to coordinate the level and form of nuclear actin with follicle development.

The mechanisms whereby PGs tightly control nuclear actin remain unknown, but there are several possibilities. First, PG regulation of cytoplasmic actin dynamics may control the pool of G-actin available for nuclear import. Supporting that such a mechanism can regulate nuclear actin, in mammalian cells, activation of integrin receptors drives cytoskeletal changes that lead to the rapid accumulation of F-actin in the nucleus (Plessner et al., 2015). Second, PG signaling could regulate the nuclear import or export of actin. Indeed, PGs regulate the actin binding protein Fascin (*Drosophila* Singed) (Groen et al., 2012) and Fascin acts with Cofilin, the cofactor for the nuclear import of actin by Imp9, to alter nuclear actin levels (Kelpsch et al., 2016). Third, PG signaling may induce the nuclear retention of actin by altering post-translational modifications on actin or its nuclear binding partners, or by controlling the levels or localization of these partners. Indeed, PGs regulate the nuclear localization of Fascin (Groen et al., 2015), making it tempting to speculate PGs regulate other nuclear actin binding proteins. Future studies will define how PGs modulate nuclear actin levels and forms.

### PG regulation of nuclear actin modifies the functions of the nucleolus

We find that PG signaling tightly controls the level and form of nuclear actin to limit rRNA transcription and protein translation, yet directly manipulating nuclear actin levels does not fully recapitulate the nucleolar phenotype in *pxt* mutants. These phenotypic differences could be due to redundant mechanism regulating nuclear actin trafficking in and out of the nucleus. Indeed, a recent study found that numerous import factors contribute to the nuclear localization of actin (Borkuti et al., 2022). Alternatively, in addition to controlling nuclear actin levels and form, PGs may also regulate the functions of actin in the nucleolus. Such regulation would remain intact when nuclear actin levels are increased in the presence of normal PG signaling.

Downstream of PG signaling, how does nuclear actin modulate nucleolar activity? One possibility is that increased nuclear actin simply increases RNAPI activity. Indeed, actin is a required cofactor for all three RNAPs, however the form of actin present in the different complex remains unclear (Kelpsch and Tootle, 2018; Green et al., 2021). Data supports that monomeric actin is bound to gene promoters and mediates RNAPII recruitment (Sokolova et al., 2018), whether this is true for RNAPI remains unknown. Actin polymerization is required for RNAPI transcription (Percipalle, 2013). Based on these findings, monomeric actin (DNase I and C4) in the nucleolus may mark and recruit RNAPI and therefore, determine which rRNA genes are transcribed, whereas polymeric actin (AC15) may function within the RNAPI complex to drive rRNA transcription. Supporting this idea, during S10B when the nurse cells are producing large quantities of mRNAs, proteins, and organelles to provide to the oocyte in S11, AC15 nucleolar puncta are observed. These puncta appear earlier in development, at an increased number, and are larger when Pxt is lost (Figures 5G–K). We speculate that these puncta are at the sites of active rRNA transcription. Monomeric actin (DNase I and C4) may also play a role in rRNA processing, as it associates with ribonucleoproteins (RNPs) (Percipalle et al., 2002; Obrdlik et al., 2008; Qi et al., 2011). RNPs splice and process 3′-ends of mRNAs. Monomeric actin also interacts with spliceosomal small nuclear RNPs and spliceosomal assembly and activation factors, and either increasing or decreasing nuclear actin impairs splicing (Viita et al., 2019). Monomeric actin remains associated with RNPs and the associated mRNA as they are transported out of the nucleus. These findings raise the possibility that nucleolar monomeric actin regulates rRNA processing and translocation to the cytoplasm. Supporting this idea, actin is present in 40S pre-mRNP/RNP ribosomal fractions (Percipalle, 2013). Together, these findings suggest monomeric nucleolar actin may regulate RNAPI activity, rRNA processing, and rRNA translocation, while polymeric nucleolar actin is required for transcription by RNAPI.

Another possibility is that nucleolar actin regulates the phase separation of the nucleolus. Nuclear actin could alter nucleolar phase separation by increasing RNAPI activity (discussed above), changing nucleolar protein-protein interactions, altering heterochromatin associated with the nucleolus, or increasing the number of actively transcribed rDNA genes (Lafontaine et al., 2021). We speculate that monomeric actin (DNase I and C4) may interact with specific nucleolar factors, driving biomolecular condensates that ultimately form and control the morphology and functions of the nucleolus. Further, monomeric actin could regulate the heterochromatin associated with the nucleolus, as it is required for the activity of multiple chromatin remodeling complexes (Kelpsch and Tootle, 2018; Green et al., 2021). Changes in chromatin architecture, along with the above discussed ability of monomeric actin to license genes for transcription, may also regulate which and how many rRNA loci are transcribed. These data lead us to hypothesize that in the context of Pxt loss, the increased nucleolar monomeric actin (DNase I and C4) alters the phase separation of the nucleolus by some or all of these mechanisms, transitioning it from a more solid-like to liquid-like state, thereby promoting rRNA transcription.

While there are many possible functions for monomeric actin in the nucleolus, one question that remains is what is the difference between DNase I and C4 monomeric actin? C4 nucleolar actin is only present in a subset of the nurse cells of any given follicle, either labeling both the anterior-most and posterior-most nurse cells or the middle nurse cells (Kelpsch et al., 2016; Wineland et al., 2018). From looking at sequential follicles within an ovariole, it seems like this C4 positivity oscillates between these two localization patterns. This spatial and temporal pattern of C4 nucleolar staining is reminiscent of what is seen for the cell cycle marker Cyclin E (Dej and Spradling, 1999), raising the possibility that C4 nucleolar actin has a cell cycle specific role in the nucleolus. The nurse cells undergo endocycling, meaning the DNA is replicated but the cell doesn’t divide. So unlike during mitosis, the nucleolus does not disassemble in the nurse cells. Thus, we speculate that C4 monomeric actin acts by an unknown means to maintain the nucleolus, and perhaps modulate nucleolar activity, during endocyling.

The literature provides many possibilities for the form-specific functions of actin within the nucleolus. Future studies are essential for determining the mechanisms whereby monomeric actin (DNase I), C4 positive monomeric actin, and AC15 positive polymeric actin modulate nucleolar function and morphology during *Drosophila* oogenesis, and whether these functions are conserved in other tissues and organisms.

### PGs, nuclear actin, nucleolar function, and oogenesis

PGs, nuclear actin, and nucleolar function are critical and conserved factors required for oocyte development and fertility. Indeed, knockout mouse models of PG synthesis and signaling components exhibit impaired follicle maturation and ovulation failure (Lim et al., 1997; Langenbach et al., 1999; Takahashi et al., 2010). Similarly, in humans, usage of non-steroidal inflammatory drugs, which inhibit COX enzymes, results in aberrant follicle maturation and delayed ovulation of fertilization incompetent oocytes (Akil et al., 1996; Smith et al., 1996; Pall et al., 2001). Nuclear actin is also emerging as a key factor in egg development. In organisms with large oocytes, like frogs and birds, actin forms a mesh or network in the nucleus that is critical for maintaining the chromatin and nucleolar distribution (Bohnsack et al., 2006; Maslova and Krasikova, 2012; Feric and Brangwynne, 2013). Nuclear F-actin is also required in mouse oocytes. There nuclear actin forms a second spindle structure to segregate chromosomes during meiosis (Mogessie and Schuh, 2017). Nuclear actin also serves to buffer the level of G-actin in the cytoplasm to prevent aberrant dense cytoplasmic F-actin networks that preclude oocyte development (Scheffler et al., 2022). Like nuclear actin and PGs, nucleolar functions, including ribosome biogenesis, play critical roles in oocytes (Mercer et al., 2021). Nucleolar activity within the oocyte is required to produce enough ribosomes to support early, preimplantation embryonic development in placental mammals and embryonic development that occurs completely outside the mother (Fulka and Aoki, 2016; Kresoja-Rakic and Santoro, 2019; Fulka et al., 2020). Further supporting the role of the nucleolus in oocyte development, mutations in human UTP14, a pre-18S rRNA processing factor, are linked to both the ribosomopathy scleroderma and infertility (Joseph et al., 2014). Thus, the nucleolus, nuclear actin, and PGs play key roles in oocyte development and fertility across organisms. This conservation of function leads us to speculate that the pathway we uncovered in *Drosophila*–where PGs tightly control the level and form of nuclear actin to coordinate nucleolar rRNA transcription and downstream protein translation rates with development to ensure the production of a high-quality oocyte–is likely to be widely used, from simple eukaryotes to humans.

## Materials and methods

For product information on the reagents used in this study please refer to Supplementary Table S1.

### Fly stocks

Fly stocks were maintained on cornmeal-agar-yeast food at 21°C. Prior to immunofluorescence or western blot analysis, flies were fed wet yeast paste daily for 3–4 days. Unless otherwise noted, *yw* (RRID: BDSC_1495) was used as the wild-type control. The following stocks were obtained from the Bloomington *Drosophila* Stock Center (Bloomington, IN): *pxt* ^*EY03052*^ (referred to as *pxt* ^*EY*^, RRID: BDSC_15620), *matαGAL4* (RRID: BDSC_7063); and UASp RNAi *actin 5C* (TRiP.HMS02487; RRID: BDSC_42651). *pxt* ^*f01000*^ (referred to as *pxt* ^*f*^) and *ebo* ^*f07537*^ (referred to as *exp6* ^*f*^) stocks were obtained from the Harvard Exelixis collection (Boston, MA). The *oskarGal4* (second and third chromosome) lines were a generous gift from Anne Ephrussi (Telley et al., 2012), and the UASp NLS-flag-Actin 5C (second and third chromosome) lines were a generous gift from Maria Vartiainen. Expression of UASp RNAi *actin 5C* was achieved by crossing to *matαGal4*, maintaining fly crosses at 21°C and maintaining progeny at 29°C for 5-6 days. Expression of UASp NLS-flag-Actin 5C was achieved by crossing to *oskarGal4* flies, maintaining fly crosses at 21°C, and maintaining progeny at 25°C for 5–6 days.

### Immunofluorescence

Whole-mount *Drosophila* ovary samples were dissected into room temperature Grace’s insect medium (Lonza, Walkersville, MD). In the cases of drug treatment, follicles were treated with either 0.4% dimethyl sulfoxide (DMSO) or 20 μg/ml Actinomycin D (Sigma-Aldrich, A1410) diluted in modified Grace’s medium containing 1X penicillin/streptomycin (100x, Gibco) and 10% fetal bovine serum (FBS, Atlanta Biologicals) for 60 min prior to antibody staining. Samples were fixed for 10 min at room temperature in 4% paraformaldehyde in Grace’s insect medium, except for the instance of methanol fixation which was done for 10 min at 4 °C in -20°C methanol. Briefly, samples were blocked by washing in antibody wash (1X phosphate-buffered saline [PBS], 0.1% Triton X-100, and 0.1% bovine serum albumin [BSA]) six times for 10 min each at room temperature. Primary antibodies were incubated overnight at 4°C, except for Actin C4, Actin AC15, and rabbit anti-fibrillarin which were incubated for a minimum of 20 h at 4°C. The following additional antibodies and concentrations were used: rabbit anti-Pxt (pre-absorbed on *pxt* mutant ovaries at 1:50 and used 1:50-1:100, (Spracklen et al., 2014b),), mouse anti-Actin C4 1:50 (RRID: AB_2223041; Millipore); mouse anti-Actin AC15 1:100 (RRID: AB_476744; Sigma-Aldrich); mouse anti-fibrillarin 72B9 1:25 (a generous gift from Patrick DiMario); rabbit anti-fibrillarin 1:250 (RRID: AB_2105785; Abcam, Cambridge, MA); and rabbit anti-flag (preabsorbed on yw ovaries at 1:20 and used at 1:20, RRID: AB_F7425; Sigma-Aldrich). After six washes in antibody wash (10 min each), secondary antibodies were incubated overnight at 4°C or for ∼4 h at room temperature. The following secondary antibodies were used at 1:250-1:500: AlexaFluor 488:goat anti-mouse (RRID: AB_2534069), AlexaFluor 568:goat anti-mouse (RRID: AB_2534072), AlexaFluor 488:goat anti-rabbit (RRID:AB_2576217), and AlexaFluor 568:goat anti-rabbit (RRID: AB_10563566) (ThermoFisher Scientific, Waltham, MA). When used, AlexaFluor488-conjugated DNase I (1:500; ThermoFisher Scientific), AlexaFluor555-or AlexaFluor657-conjugated Phalloidin (1:500; ThermoFisher Scientific) and AlexaFluor555-or AlexaFluor647-conjugated wheat germ agglutinin, WGA (1:500; ThermoFisher Scientific) were included with the primary and secondary antibodies. Following six washes in antibody wash (10 min each), 4′,6-diamidino-2-phenylindole (DAPI, 5 mg/ml) staining was performed at a concentration of 1:5,000 in 1X PBS for 10 min at room temperature. Samples were then rinsed in 1X PBS and mounted in 1 mg/ml phenylenediamine in 50% glycerol, pH 9 (Platt and Michael, 1983). For each experiment ∼5 pairs of ovaries were stained, imaged, and analyzed, and all experiments were performed a minimum of three independent times.

### Click-iT nascent RNA staining

Whole mount *Drosophila* ovary samples (5-10 ovaries per sample) were either treated with 0.2-2 μg/ml of Actinomycin D diluted in Grace’s medium, 250 μg/ml Alpha-amanitin (Sigma-Aldrich, A2263) in Grace’s medium or the same dilution of DMSO for 10 min prior to, or were directly incubated with 2 mM 5-ethynyl uridine (EU) for 15 min and washed twice with Grace’s medium. Samples were fixed in 4% paraformaldehyde diluted in Grace’s medium for 15 min. Samples were washed 6 times for 10 min each with Triton Wash (1X PBS, 0.5% Triton X-100), and then rinsed once with 1X PBS before incubating for 30 min in the Click-iT reaction cocktail, prepared according to product specifications (Thermo Fisher Scientific, Click-iT™ RNA Alexa Fluor™ 488 Imaging Kit, C10329). Samples were rinsed twice with Click-iT Reaction Rinse Buffer and twice with 1X PBS, and then washed for 30 min in 1X PBS. Samples were either stained for antibodies starting at the antibody wash step (see above) or immediately stained with Hoechst 33342 (1:1000 in PBS) for 15 min to label DNA and then rinsed 3 times in 1X PBS before mounting as described in the immunofluorescence section.

### Image acquisition and processing

Microscope images of fixed *Drosophila* follicles were obtained using Zen software on a Zeiss 880 mounted on Zeiss Axio Observer.Z1 using Plan-Apochromat 20x/0.8 working distance (WD) = 0.55 M27, Plan-Apochromat 40x/1.3 Oil DIC WD = 2.0, or Plan-Apochromat 63x/1.4 Oil DIC f/ELYRA objectives (Carl Zeiss Microscopy, Thornwood, NY), on a Zeiss 700 mounted on an Axio Observer.Z1 using a Plan-Aprochromat 20x/0.8 WD = 0.55 M27 (Carl Zeiss Microscopy, Thornwood, NY) or LAS AF SPE Core software on a Leica TCS SPE mounted on a Leica DM2500 using an ACS APO 20×/0.60 IMM CORR -/D or an ACS APO 63×/1.30 Oil CS 0.17/E objective (Leica Microsystems, Buffalo Grove, IL). Maximum projections, merged images, rotation, and cropping were performed using ImageJ software (Abramoff et al., 2004, FIJI, RRID: SCR_002285). All fluorescent images were brightened by 30% in Photoshop (Adobe, RRID: SCR_010279) to improve visualization, except Figure 1; Supplementary Figures S1, S4 where the images were brightened by 50%.

### Quantification of nuclear actin from immunofluorescence

Genotypically de-identified images were analyzed using ImageJ (Abramoff et al., 2004) for DNase I, C4 and AC15 for specific stages of oogenesis. Follicle staging was assigned based on morphology and size.

DNase I nucleolar to cytoplasmic ratios were quantified from single confocal slices in S7/8 follicles by measuring the integrated density of fluorescence within a square in the nucleolus, compared to a square in the adjacent cytoplasm; the focal planes chosen had the strongest nucleolar DNase I signal. Three paired measurements were made per cell and the average nucleolar/cytoplasmic ratio was determined. Three cells per follicle were measured. DNase I data were analyzed and statistical analysis performed using Prism (Graphpad, RRID: SCR_002798).

Quantification of C4 nucleolar actin was performed on confocal image stacks of follicles stained with anti-actin C4, WGA, and Phalloidin; as necessary, brightness and contrast were adjusted to score all the C4 nucleolar actin present. Data were collected for S5-6, S7-8, and S9 follicles. For each follicle the number of nurse cells exhibiting structured nucleolar C4 actin was scored.

Quantification of AC15 nuclear actin level and nucleolar puncta presence/size was performed on confocal image stacks of follicles stained with anti-actin AC15, WGA, and DAPI. For AC15 nuclear actin level, data were collected for S5-6, S7-8, and S9 follicles. For each follicle the nurse cells and the follicle cells were scored for their level of AC15 staining on a 5-point scale ranging from background levels typical of what is observed in wild-type S3 follicles (-) to the strongest staining typical of what is observed in wild-type S10 follicles (+++). For AC15 puncta, data were collected for S7/8, S9, and S10 follicles; as necessary, brightness, contrast and zoom were adjusted to score the puncta. Each follicle was scored as having either no AC15 puncta, small puncta, or large/obvious puncta in the nurse cells. C4 and AC15 data were analyzed using Excel (Microsoft, RRID: SCR_016137) and statistical analysis was preformed using R (Vienna, Austria, RRID: 001905).

### Quantification of nucleolar morphology

Confocal image stacks of S10B follicles stained for fibrillarin or EU were genotypically blinded and each follicle was assigned a nucleolar defect severity score according to the number of nurse cell nucleoli with morphological changes; the four posterior nurse cells were excluded from the analysis as their morphology is distinct. The nucleolar morphology categories included: normal (0-1 disrupted nucleoli), mild (2-4 disrupted nucleoli), and severe (5 or more disrupted nucleoli). Morphology defects included nucleoli that are pebbled (several smaller, disconnected portions of nucleolus) or rounded (single, nearly spherical nucleolus). In the context of NLS Actin overexpression (Figure 6), we assessed nucleolar morphology in relation to the level of nuclear actin. The level of nuclear actin was categorized as low which exhibits a nuclear actin haze, medium which exhibits a network of thin nuclear actin filaments, and high which exhibits thick nuclear actin filamentous structures termed rods. Quantification of nucleolar volume was performed using Imaris (Oxford, RRID:SCR_007370) on *yw* S10B follicles stained for EU. The single anterior-most nurse cell was compared to the average volume of four posterior nurse cells (from the third row of nurse cells).

### Quantification of Click-iT nascent RNA staining

Click-iT EU intensity was quantified from genotypically de-identified confocal image stacks of S10 follicles. For a given follicle, the EU intensity of three nurse cells was measured, excluding the four posterior-most and single anterior-most nurse cells. For each nurse cell, three nucleolar and three cytoplasmic intensity measurements of a square ROI (4.788 square pixels) were taken and averaged. The average cytoplasmic intensity measurement was subtracted from the averaged nucleolar intensity measurement, resulting in a single nucleolar relative EU intensity value per nurse cell.

### Puromycin incorporation assay

From 1-2 day old flies, 10-15 ovary pairs were dissected per sample in Grace’s insect medium. Samples were transferred to a 9-well plate containing 5 μg/ml puromycin dihydrochloride (Sigma-Aldrich) diluted in Grace’s medium and incubated with shaking for 40 min. Samples were then washed with 1X PBS and then transferred to 1.5 ml microcentrifuge tubes. The PBS solution was removed from sample tubes and 50 μL 1X Laemmli buffer was added. The samples were ground by hand with a plastic pestle, and then boiled for 10 min. Samples were stored at -20°C until used for western blotting.

### Western blotting

Whole ovaries were dissected from flies fed wet yeast paste for 2-3 days, unless otherwise noted. Ovaries were dissected in room temperature Grace’s insect medium and transferred to a 1.5 ml microcentrifuge tube containing 50 µL of Grace’s media. Grace’s media was removed and replaced with 50 µL 1X Laemmli buffer. The tissue was lysed by grinding with a plastic pestle and boiled for 10 min. Samples were run on a 10% SDS-PAGE gel; in some cases, initial western blot analysis was used to dilute samples to normalize protein levels. The molecular weight ladder used in all experiments was the Precision Plus Protein All Blue Prestained Protein Standard (BioRad, 1610373). Western blots were performed using standard methods (Biorad system). For the puromycin incorporation experiments, prior to blocking and antibody incubation blots were stained with 0.1% Ponceau S solution in 1% acetic acid for 10 min and photographed using an iPhone 13 Pro; Ponceau S solution was washed off prior to antibody stains. The following primary antibodies were obtained from the Developmental Studies Hybridoma Bank (DSHB) developed under the auspices of the National Institute of Child Health and Human Development and maintained by the Department of Biology, University of Iowa (Iowa City, IA): mouse anti-Puromycin 0.5ug/mL (MY-2A4, Yewdell, J, (David et al., 2012)) and rat anti-vasa 1:100 (vasa, Spradling, A.C/Williams, D, (Aruna et al., 2009)). The following primary antibodies were also used: mouse anti-actin AC15 1:200, rabbit anti-RpL11 1:1000 (RRID:AB_2042832, Abcam, Cambridge, MA), mouse anti-RpS6 1:500 (RRID: AB_2238583, Cell Signaling), rabbit anti-Exp6 1:50,000 (Yves, a generous gift from Dirk Goerlich (Stuven et al., 2003)), and mouse anti-alpha tubulin 1:5000 (RRID: AB_477593, Sigma-Aldrich). All blots were blocked and incubated in primary antibody in 5% milk diluted in 1X Tris-buffered saline (1X TBS) with 0.1% Tween 20, except for anti-actin AC15, anti-RpS6, anti-RpL11, and anti-Exp6, blots which were blocked and incubated in primary antibody in 3% BSA diluted in 1X TBS with 0.1% Tween 20. The following secondary antibodies were obtained from Jackson ImmunoResearch Laboratories (West Grove, PA) and used 1:5,000: Peroxidase-AffiniPure Goat Anti-Mouse IgG (H + L) (RRID: AB_10015289), Peroxidase-AffiniPure Goat Anti-Rabbit IgG (H + L) (RRID: AB_2307391), and Peroxidase-AffiniPure Goat Anti-Guinea Pig (H + L) (RRID: AB_2337402), except for the anti-RpS6 and anti-RpL11 western blots where secondary antibodies were used at 1:10,000. Blots were developed with SuperSignal West Pico or Femto Chemiluminescent Substrate (Thermo Scientific, Waltham, MA) and imaged using the Amersham Imager 600 (GE Healthcare Life Sciences, Chicago, IL). A minimum of three independent experiments were performed for each Western blotting experiment. Where not shown as full blots in the figures, full blots are provided in Supplementary Figure S8.

## Data Availability

The original contributions presented in the study are included in the article/Supplementary Material, further inquiries can be directed to the corresponding author.
