# Supplementary material for: Prostaglandins limit nuclear actin to control nucleolar function during oogenesis
Source: Front Cell Dev Biol. 2023 Feb 17;11:1072456. doi: 10.3389/fcell.2023.1072456 (PMC9981675; doi:10.3389/fcell.2023.1072456)
Supplement: Supplementary file 2 [file DataSheet1.pdf]

Supplemental Figures

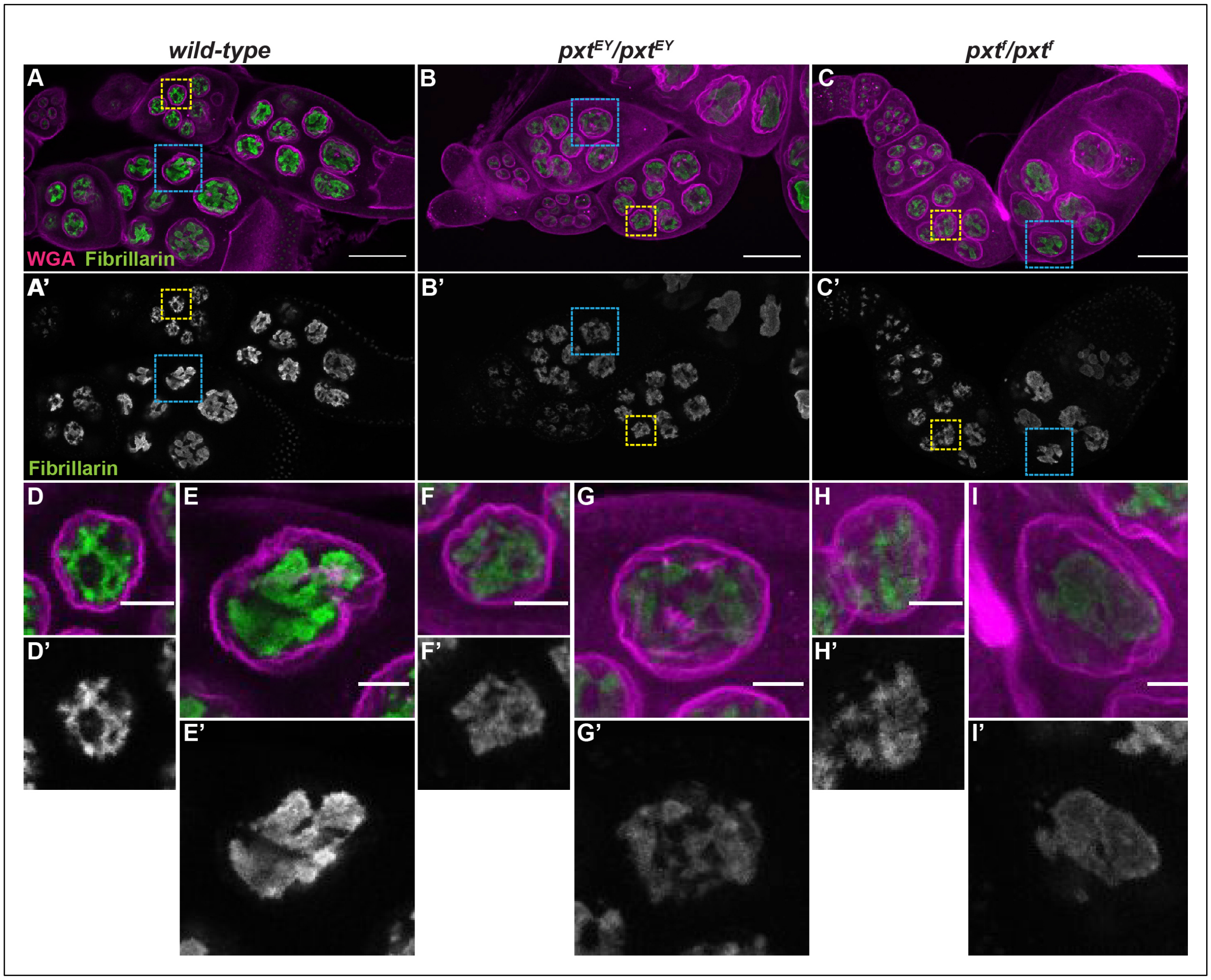

SFigure 1: PGs regulate nucleolar morphology during early oogenesis. A-I'. Maximum projections of 3-4 confocal slices of wild-type (*yw*) and *pxt* mutant (*pxt*$^{EY/EY}$ and *pxt*$^{f/f}$ as indicated) follicles stained for: nucleolus (Fibrillarin, green in merge and white in single channel) and nuclear envelope (WGA, magenta in merge). Yellow boxed regions in **A-C'** (S6/7) are shown as zoomed in images in **D-D'**, **F-F'**, and **H-H'**, and blue boxed regions in **A-C'** (S9) are shown as zoomed in mages in **E-E'**, **G-G'**, and **I-I'**. In **A-C'**, scale bars = 50µm, in **D-I'** scale bars = 10µm. In early stages (S6/7 and earlier), the nucleoli exhibit a thin reticular pattern (**A-A'**, **D-D'**); loss of Pxt results in a more diffuse nucleoli that have a less clear structure (**B-C', F-F'** and **H-H'**). By S9, in wild-type follicles the nucleoli have an interconnected tubular morphology (**E-E')**. Loss of Pxt results in thinner, more diffuse tubules (**G-G'**) or decreased tubular structure that becomes more rounded (**I-I'**).

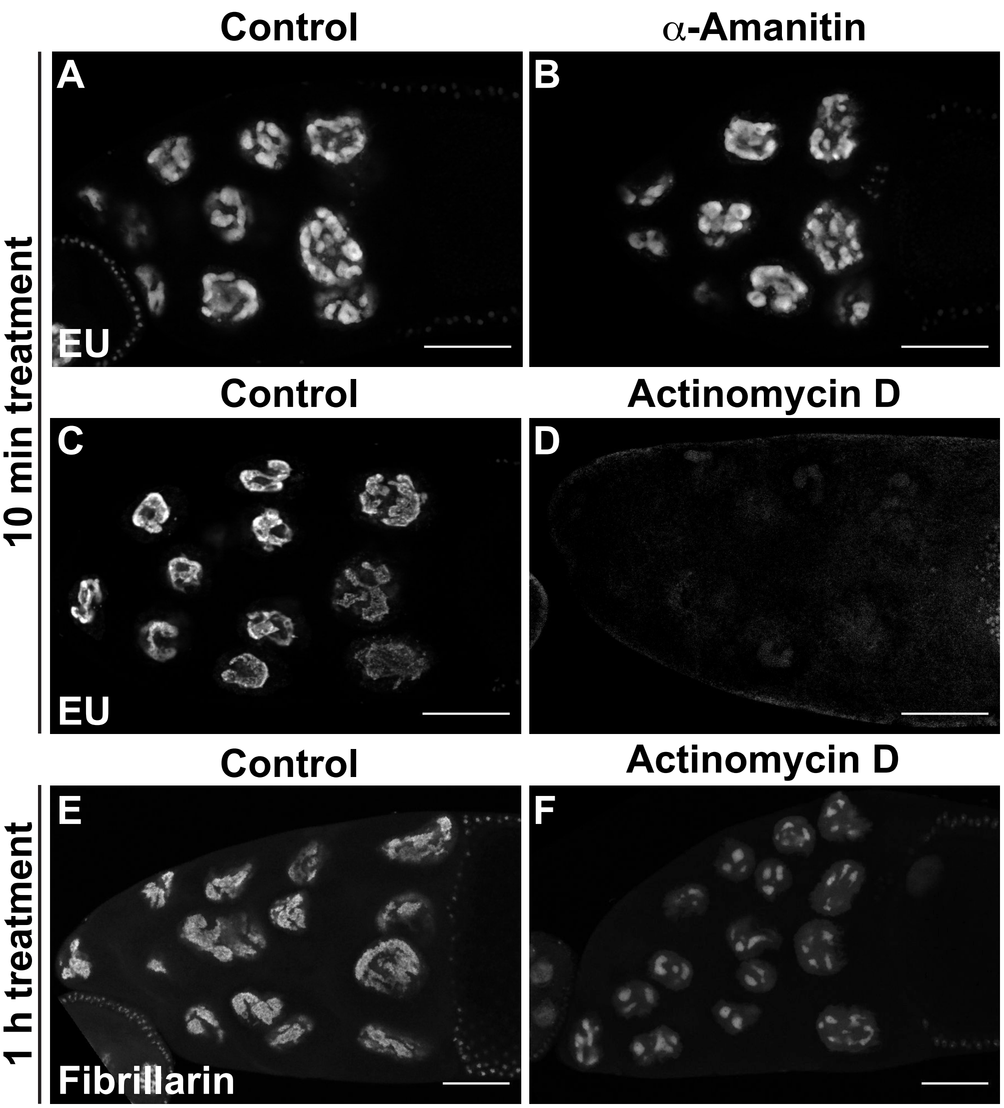

SFigure 2: Nascent nucleolar RNA and nucleolar morphology controls. A-D. Maximum projections of 2 confocal slices of Control (DMSO treated) and drug (**B** = 250μl/ml α-amanitin and **C** = 20μg/ml Actinomycin D) 10 min-treated S10B follicles stained for nascent RNA (EU). **E-F.** Maximum projections of 2 confocal slices of Control (DMSO treated) and 20μg/ml Actinomycin D 1 hr-treated S10B follicles stained for the nucleolus (Fibrillarin). Scale bars = 50μm. Inhibition of RNAPII (**A-B**) has no gross effect on nascent nucleolar RNA, whereas inhibition of RNAPI blocks nucleolar transcription (**C-D**). Blocking RNAPI activity results in tubular nurse cell nucleoli (**E**) fragmenting into multiple small, round nucleoli (**F**).

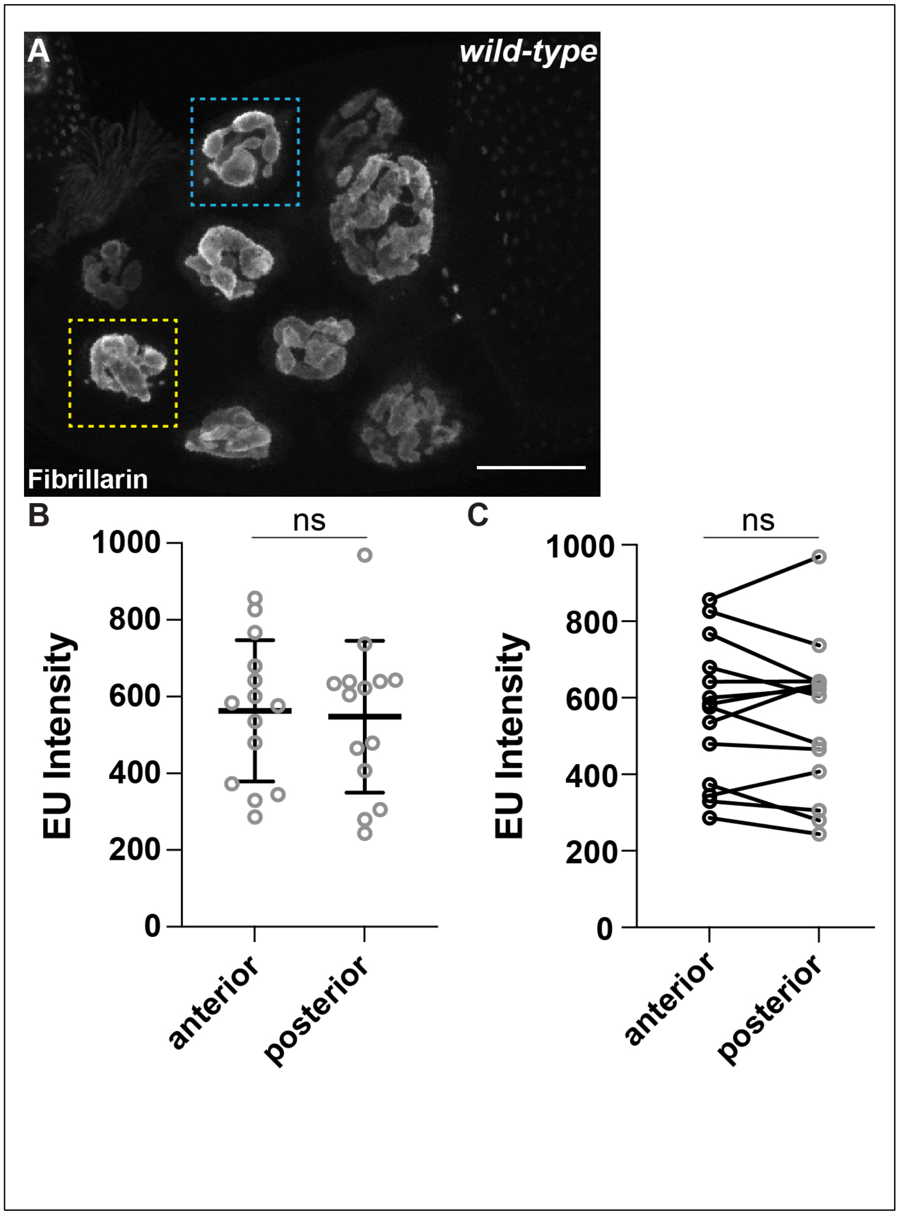

SFigure 3: Nucleolar volume does not impact EU intensity. A. Maximum projections of 5 confocal slices of wild-type (*yw*) S10B follicle stained for nucleolus (Fibrillarin). Yellow box indicates an example of an anterior nurse cell nucleolus and blue box indicates an example of a posterior nurse cell nucleolus. Scale bars = 50µm**. B-C.** Graphs quantifying the anterior vs posterior nurse cell nucleolar EU intensity. In **B**, the average value is shown (unpaired, two-tailed t-test, p=0.47), whereas in **C** the paired data are shown (paired t-test, p=0.83). The nurse cells within a follicle exhibit distinct differences in size, including their nucleoli, with the size increasing from anterior to posterior (**A**). Even though anterior nucleoli are smaller in volume, the EU intensity (nascent rRNA levels) is not different between anterior and more posterior nurse cells (**B-C**).

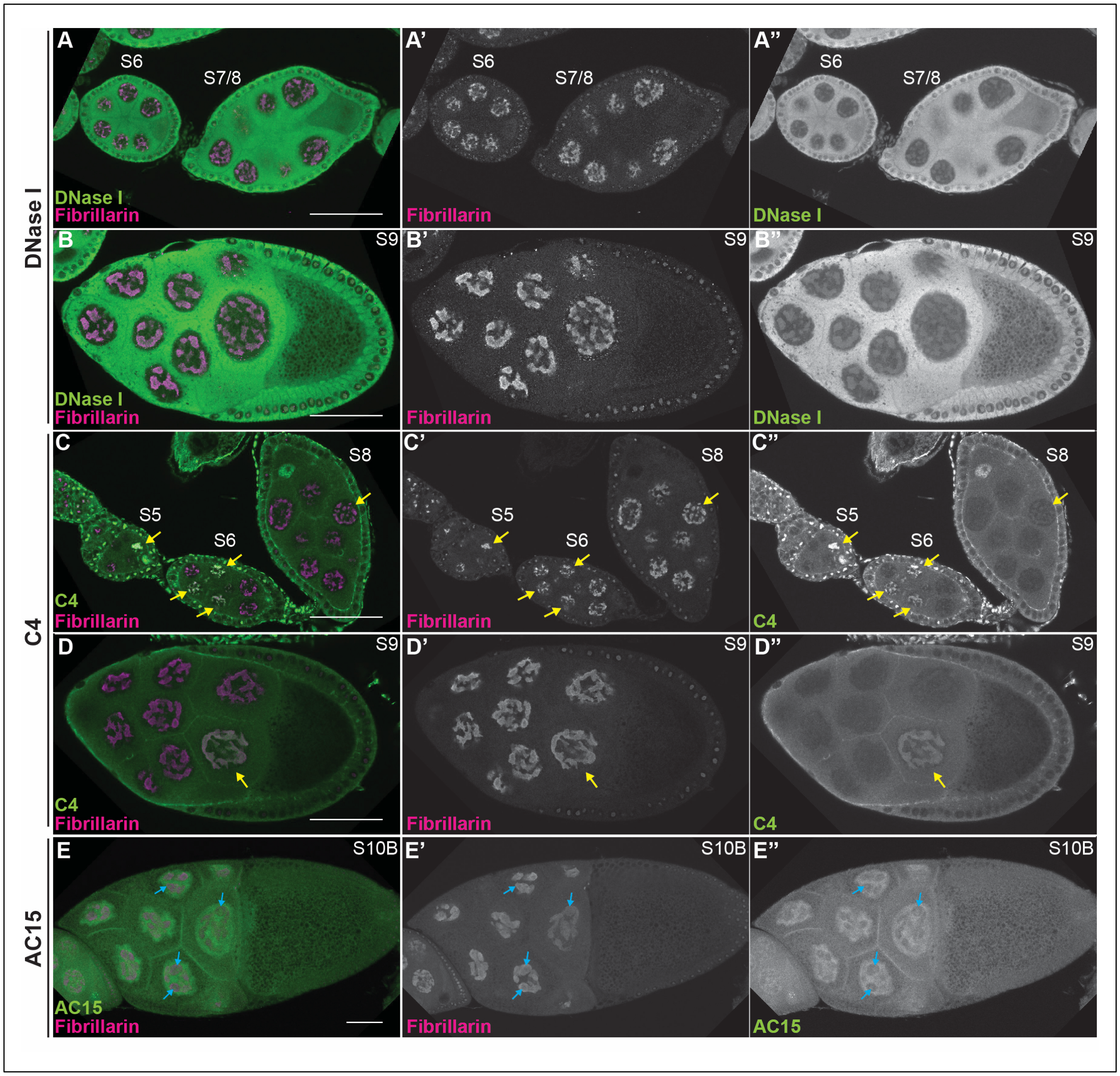

SFigure 4: Nuclear actin is enriched in the nucleolus. A-E". Maximum projections of 2-5 confocal slices of wild-type follicles of the indicated stages (S) stained for the nucleolus (Fibrillarin, magenta in merge) and three different nuclear actin labeling tools (green in merge): DNase I (**A-B"**), anti-actin C4 (C4, **C-D"**), and anti-actin AC15 (AC15, **E-E"**); scale bars = 50µm. Yellow arrow indicates a C4 positive nucleoli and blue arrows indicate examples of AC15 positive nucleolar puncta. Note that all images were brightened 50% in photoshop to improve clarity and the images shown in Figure 3A-C" are zoomed in images of B-B", D-D", and E-E". DNase I, which labels all monomeric actin is enriched throughout the nucleolus of every cell across all of oogenesis (**A-B"**), whereas C4 labels whole nucleoli of a subset of nurse (yellow arrows) and follicle cells from S3-S9 (**C-D"**). AC15 nuclear actin is largely localized to the chromatin, but in mid-oogenesis it labels puncta within the nurse cell nucleoli (**E-E"**, blue arrows).

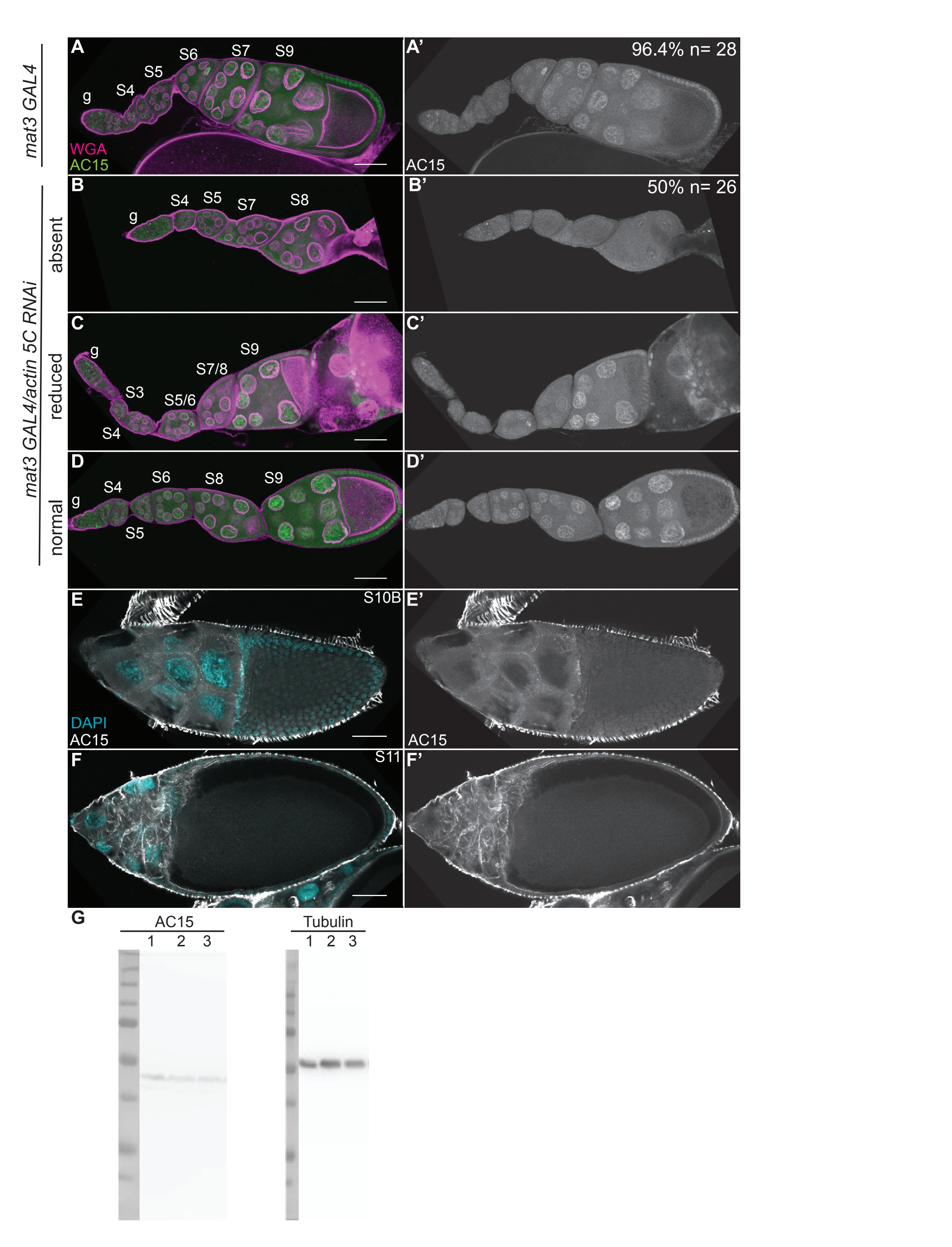

SFigure 5: Assessing the specificity of the AC15 antibody. A-D'. Maximum projections of 2-4

confocal slices of *mat 3 GAL4* (control) and *mat 3 GAL4/actin 5C RNAi* (experimental) follicles of the indicated stages (S, germarium = g) stained for: nuclear envelope (WGA, magenta in merge) and anti-actin AC15 (AC15, green in merge). The follicles were scores for AC15 staining as being absent, reduced or normal; the percentages are noted in **A'** and **B'**. **E-F'**. Maximum projections of 2-4 confocal slices of wild-type follicles of the indicated stages that were fixed in ice-cold methanol and stained for AC15. **G.** Western blots of three wild-type whole ovary lysates stained for anti-actin AC15 and tubulin (control). To validate the specificity of the AC15 antibody for actin, we used RNAi to weakly knockdown the expression of *actin 5C* in the germline; this results a reduction in nuclear AC15 labeling compared to controls (**A-D'**). Knockdown was verified by reduced phalloidin staining and late-stage follicle morphogenesis defects (data not shown). Additionally, to verify that the AC15 antibody can label F-actin, we used methanol fixation to make the actin within filaments more accessible to the antibody. We find that the AC15 antibody no longer labels nuclear actin, but instead labels all canonical F-actin structures (**E-F'**). Finally, we examined the specificity of the AC15 antibody using immunoblot and find that it labels one strong band at the molecular weight of actin and a weak band of similar size (**G**); this raises the possibility that it may also recognize a modified form of actin. Together, these findings support that the AC15 antibody specifically recognizes nuclear actin using our standard immunofluorescence approach. Scale bars = 50μm.

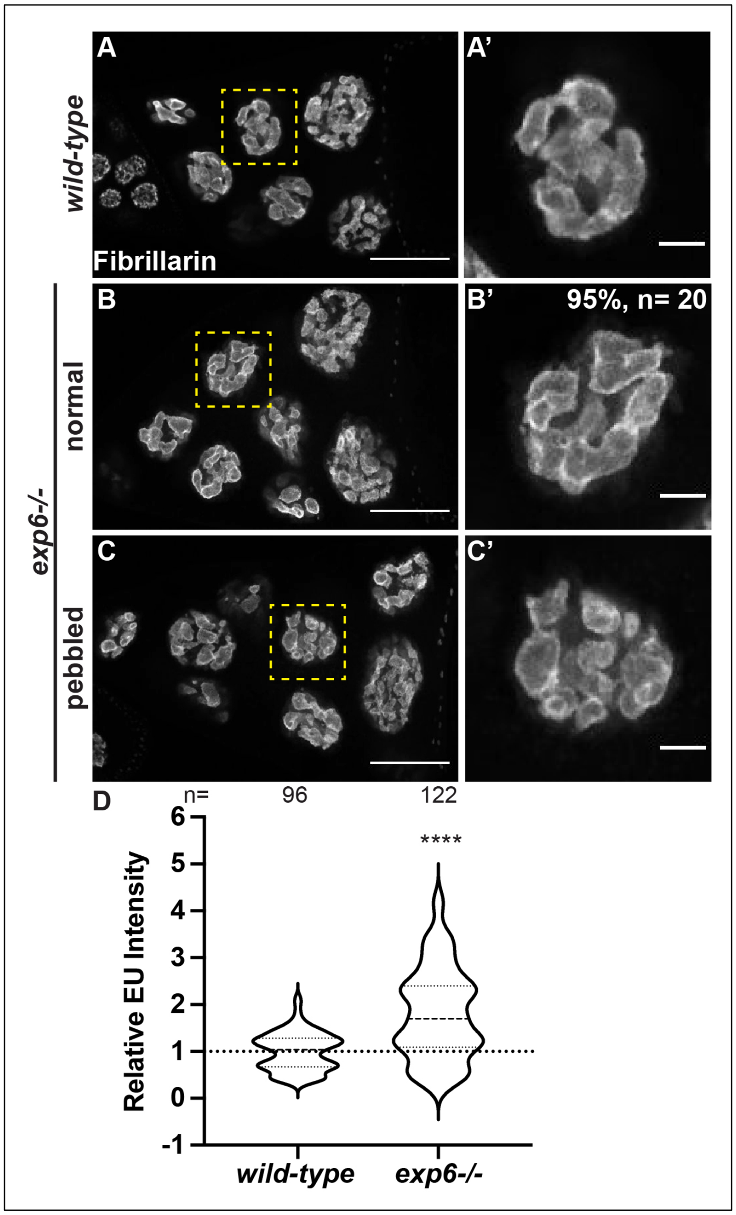

SFigure 6: Exportin 6 limits nucleolar activity. A-C’. Maximum projections of 2 confocal slices of wild-type and *exp6-/-*S10B follicles (**A-C**, scale bars = 50µm) or zoomed in images of single nurse cell nucleoli boxed in yellow (**A’-C’**, scale bars = 10µm) stained for the nucleolus (Fibrillarin). Examples of the varying nucleolar morphologies in the *exp6-/-* follicles are shown, normal and pebbled, n= number of follicles. **D.** Graph quantifying the relative EU fluorescence intensity of the indicated genotypes, ****$p<0.0001$ (unpaired t-test). n = number of follicles. While there is not a statistical difference in nucleolar morphology phenotypes when Exp6 is lost (data not shown), the mutants do exhibit some defects, including a pebbled nucleolar morphology (**C-C’**, compared to **A-B’**). However, loss of Exp6 results in increased nascent nucleolar RNA (**D**).

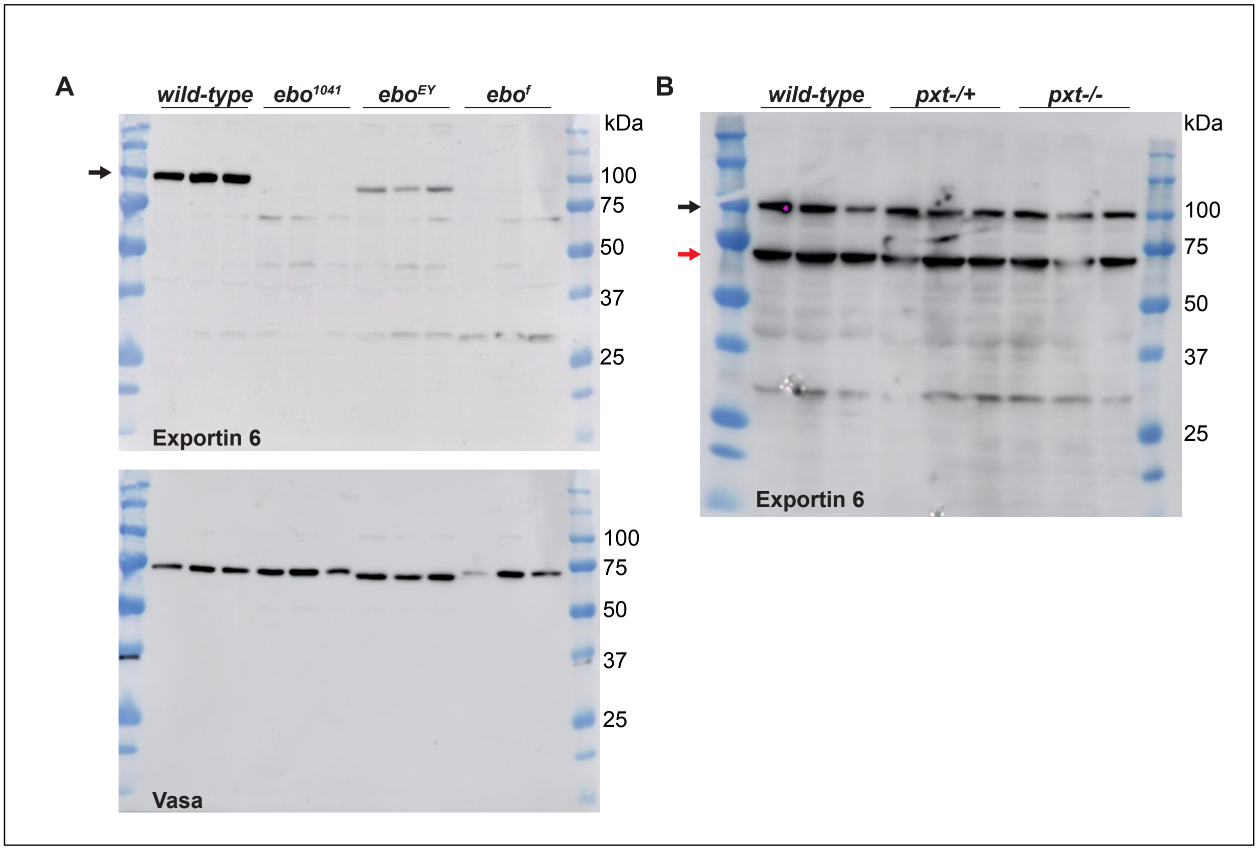

SFigure 7: Western blots for Exportin 6. A. Western blots of whole ovary lysates of wild-type and *exp6* alleles stained for Exportin 6 (Exp6, black arrow) and Vasa (loading control). The $exp6^f$ was used in the data presented throughout the paper. **B.** Western blot of wild-type (yw), pxt^{EY}/+ (*pxt-/+*) and pxt^{EY}/pxt^{EY} (*pxt-/-*) whole ovary lysates stained for Exp6, the Exp6 band is indicated with the black arrow and the background band (loading control) is indicated with the red arrow. Molecular weight ladder is BioRad Precision Plus Protein Standard. The $exp6^f$ allele used in these studies is a strong hypomorph, with little to no protein present **(A).** Loss of Pxt does not alter Exp6 protein levels (**B**).

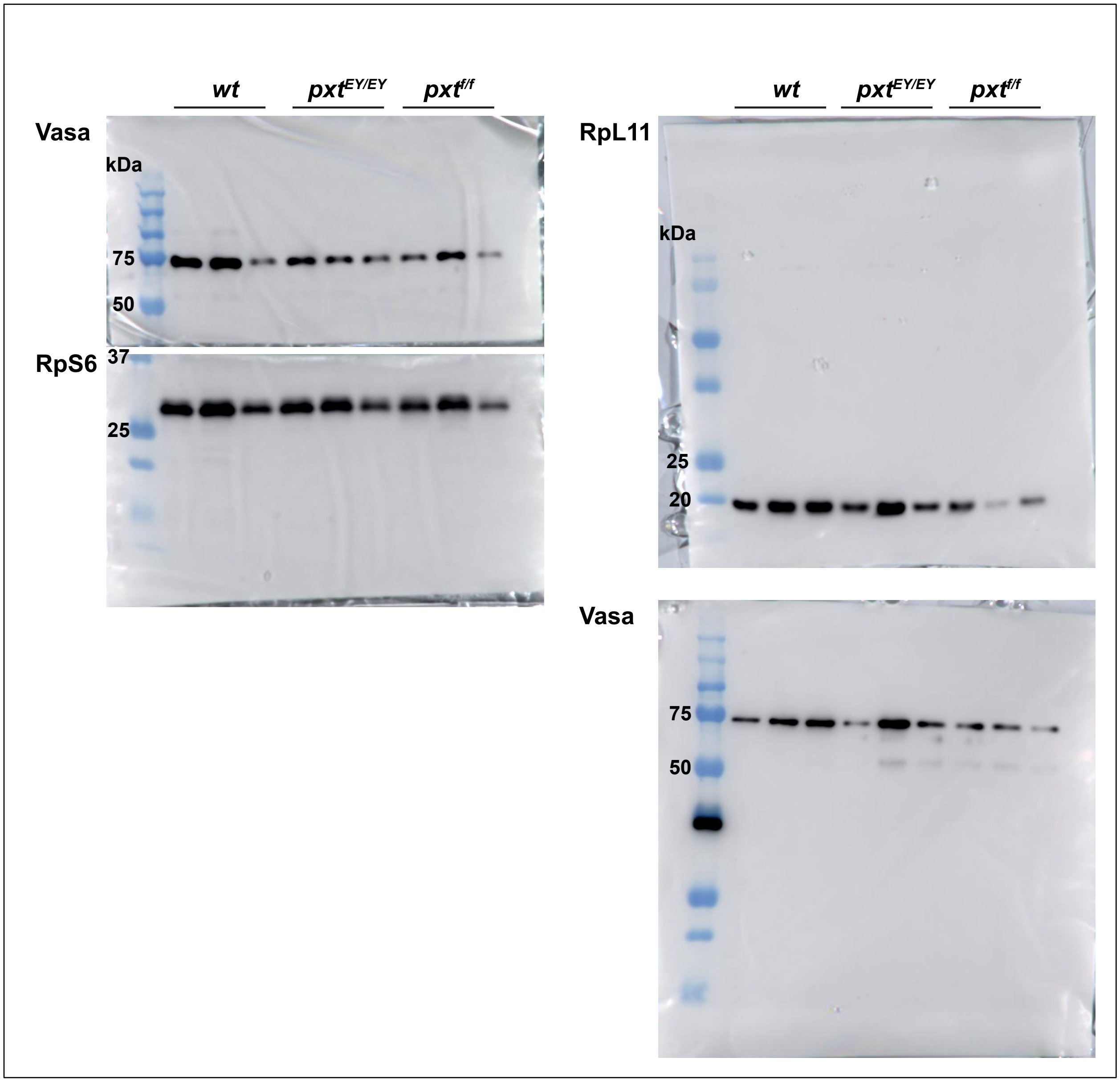

SFigure 8: Whole western blots. Whole western blots from Figure 2A stained for RpS6, RpL11, and Vasa (loading control). On the left are images of the blot cut horizontally at the 37kDa marker band, with the top probed for Vasa and the bottom probed for RpS6. On the right are 2 blots loaded with the same samples; the top is probed for RpL11 and the bottom for Vasa (loading control). Molecular weight ladder is BioRad Precision Plus Protein Standard.

Movie 1: AC15 labels puncta within the nurse cell nucleoli. Movie of the confocal stack of a region of S10B follicle stained for the nucleolus (Fibrillarin, magenta in merge) and AC15 (green in merge), single channels are shown in white. Note that this is the same follicle shown in Figure 3C-C' and SFigure 4E-E". Scale bars = 50µm. AC15 labels puncta that reside inside the Fibrillarin-labeled nucleoli.

STable 1: Key Resources Table

STable 2: Raw Data Table. Raw data and statistics used for all quantifications presented in both the primary and supplemental figures.
